# Genome and whole-genome resequencing of *Cinnamomum camphora* elucidate its dominance in subtropical urban landscapes

**DOI:** 10.1186/s12915-023-01692-1

**Published:** 2023-09-12

**Authors:** Danqing Li, Han-Yang Lin, Xiuyun Wang, Bo Bi, Yuan Gao, Lingmei Shao, Runlong Zhang, Yuwei Liang, Yiping Xia, Yun-Peng Zhao, Xiaofan Zhou, Liangsheng Zhang

**Affiliations:** 1 Genomics and Genetic Engineering Laboratory of Ornamental Plants, College of Agriculture and Biotechnology, Zhejiang University, Hangzhou, China; 2 Laboratory of Systematic and Evolutionary Botany and Biodiversity, College of Life Sciences, Zhejiang University, Hangzhou, China; 3 School of Advanced Study, Taizhou University, Taizhou, China; 4 ZJU-Hangzhou Global Scientific and Technological Innovation Center, Hangzhou, China; 5 Shenzhen Branch, Guangdong Laboratory of Lingnan Modern Agriculture, Genome Analysis Laboratory of the Ministry of Agriculture and Rural Affairs, Agricultural Genomics Institute at Shenzhen, Chinese Academy of Agricultural Sciences, Shenzhen, China; 6 Guangdong Laboratory for Lingnan Modern Agriculture, Guangdong Province Key Laboratory of Microbial Signals and Disease Control, Integrative Microbiology Research Center, South China Agricultural University, Guangzhou, China; 7 Hainan Institute of Zhejiang University, Sanya, China

**Keywords:** *Cinnamomum camphora*, Evergreen broadleaved tree, Comparative genomics, Whole-genome resequencing, Dominance, Environmental adaptation

## Abstract

**Background:**

Lauraceae is well known for its significant phylogenetic position as well as important economic and ornamental value; however, most evergreen species in Lauraceae are restricted to tropical regions. In contrast, camphor tree (*Cinnamomum camphora*) is the most dominant evergreen broadleaved tree in subtropical urban landscapes.

**Results:**

Here, we present a high-quality reference genome of *C. camphora* and conduct comparative genomics between *C. camphora* and *C. kanehirae*. Our findings demonstrated the significance of key genes in circadian rhythms and phenylpropanoid metabolism in enhancing cold response, and *terpene synthases* (*TPSs*) improved defence response with tandem duplication and gene cluster formation in *C. camphora*. Additionally, the first comprehensive catalogue of *C. camphora* based on whole-genome resequencing of 75 accessions was constructed, which confirmed the crucial roles of the above pathways and revealed candidate genes under selection in more popular *C. camphora*, and indicated that enhancing environmental adaptation is the primary force driving *C. camphora* breeding and dominance.

**Conclusions:**

These results decipher the dominance of *C. camphora* in subtropical urban landscapes and provide abundant genomic resources for enlarging the application scopes of evergreen broadleaved trees.

**Supplementary Information:**

The online version contains supplementary material available at 10.1186/s12915-023-01692-1.

## Background

Lauraceae from the order Laurales within Magnoliids, which contains 45 genera of a total of 2500–3000 species, represents an early evolutionary lineage of flowering plants (angiosperms) [1, 2]. Members of this family have attracted attention due to their crucial phylogenetic position and important economic and ornamental value [1, 3–5]. Most evergreen species in Lauraceae are restricted to tropical regions and are especially diverse in Asia and the Americas [2, 6]. Intriguingly, camphor tree (*Cinnamomum camphora*) is native to East Asia and extensively distributed throughout the major tropical and subtropical regions and the transition zone between subtropical and temperate climates [7, 8]. It is one of the most famous ornamental trees with evergreen broadleaf and has dominance in subtropical urban landscapes [9]. Moreover, *C. camphora* fixes a large amount of CO_2_ and interacts with the environment, playing an important role in carbon fixation and global climate changes. There characteristics make *C. camphora* ecologically important. Ongoing attempts were made to expand the application scope of *C. camphora* on account of its significant value for timber, aromatic products, urban landscapes, and ecological environment in recent years [10–12]; however, the evolution and mechanisms underlying *C. camphora*’s dominance in subtropical urban landscapes compared with other species of the *Cinnamomum* L. genus, or even in Lauraceae, have not been deciphered previously.

In contrast to the widespread distribution of *C. camphora*, its relative *C. kanehirae* is restricted in broadleaved forests, mainly in Taiwan, China, and faces the threat of extinction [3]. The divergent distribution renders *C. camphora* and *C. kanehirae* a fantastic system to elucidate the mechanisms related to their contrasting environmental adaptation. The ability of environmental adaptation depends on the response to abiotic stress, such as extreme temperatures, droughts, or food shortages, and biotic stress, such as fungi, bacteria, and insects [13, 14]. Previous studies have confirmed that the growth of *C. kanehirae* is sensitive to air temperatures [15], which may lead to its restricted distribution. In contrast, *C. camphora* could withstand temperatures as low as −7 ℃. Furthermore, the components of volatile oils, mostly terpenoids and their derivatives, are derived from *Cinnamomum* species [16, 17]*.* These terpenoids are not only the main constituents of essential oils but also play an important role in the defence response against biotic stress [18–20]. Terpene synthases (TPSs) is responsible for the synthesis of the various terpenoids by two independent pathways: the 2-C-methyl-D-erythritol 4-phosphate (MEP) pathway in plastids and the mevalonate (MVA) pathway in the cytosol, respectively. According to the phylogenetic relationships, plant TPSs can be categorized into seven distinct clades or subfamilies, including a, b, c, d, g, e/f, and h. Of these, TPS-a, TPS-b, and TPS-g clades are specific in the angiosperm, which comprise entirely of genes of specialized mono-, sesqui-, or diterpene biosynthesis. Despite the large expansion of *TPS* gene family in *C. kanehirae* [3] and the identification of TPSs in *C. camphora* [10, 21–24], the functional divergence of genes in terpene biosynthesis and defence response pathways are still mysteries. Thus, a genome-wide comparison between *C. camphora* and *C. kanehirae* will facilitate a better understanding of the evolutionary adaptation to both abiotic and biotic stress in *Cinnamomum*.

Recent studies have provided reference genomes for *C. camphora* [21–24]; however, the genome quality still needs improvements to precisely identify genes and variations underlying important selected traits. For example, the gene numbers of TPS family in these studies varied, especially those in functionally important TPS-b clade. Besides, the current *C. camphora* genome studies mainly emphasize the biosynthesis of aromatic compounds, rather than environmental adaptations, which determine plant distribution and quality. *C. camphora* individuals exhibit a wide range of diversity in aromatic compounds, stress tolerance, and other important traits [25]. This diversity might be the result of evolutionary diversification that drove strong adaptation throughout the tropical and subtropical regions in East Asia. Overall, characterization of genome diversity and discovery of genes controlling environmental adaptation by functional genomic and population genetic approaches will help to uncover the reasons for the dominance of *C. camphora* in subtropical urban landscapes.

To reveal the genetic basis of the distinct environmental adaptation in closely related *Cinnamomum* species, we provide a high-quality genome of *C. camphora* at the chromosome level using Pacific Biosciences (PacBio) sequencing and high‐throughput chromosome conformation capture (Hi-C) technology. Comparative genomic analyses between *C. camphora* and *C. kanehirae* were performed to elucidate their microevolutionary differences. Moreover, genomic resources, such as single-nucleotide polymorphisms (SNPs), could lead to the identification of loci underlying genetic diversity [26], which are possibly the result of the evolutionary trajectory that drove the dominance of *C. camphora* in subtropical urban landscapes. We also present analyses of whole-genome resequencing of 75 individuals of *C. camphora* and provide the first comprehensive database of molecular variation in this species. In essence, the comparative genomics and whole-genome resequencing analyses in the present study discovered genes controlling cold adaptability and important agronomic traits in *C. camphora*, revealed the reason for its dominance in subtropical urban landscapes, and accelerated genetic improvement in the *Cinnamomum* genus and other evergreen trees.

## Results

### Genome assembly, annotation, and quality assessment

To assemble the genome of *C. camphora*, a combination of Illumina short-read sequencing and PacBio long-read sequencing technology was applied. The sequencing of *C. camphora* resulted in coverage of ~161.65-fold PacBio single-molecule long reads (111.06 Gb with an average length of 18,420 bp), and 72.19-fold Illumina paired-end short reads (49.59 Gb). An initial assembly of PacBio sequencing data was generated using Canu v2.0, which was subsequently processed using purge_dups v1.0.1 and polished using NextPolish v1.3.1, yielding 205 contigs (total assembly size: 680.79 Mb; N50: ~9.18 Mb). Then, RaGOO [27] was used to anchor the scaffolds to the *C. kanehirae* reference chromosomes. Additionally, we constructed Hi-C libraries of *C. camphora* (Additional file 2: Fig. S1), generating 71.00 Gb of Hi-C paired-end reads to verify the results of chromosome allocation. This enabled 97.90% of the assembled sequences to be anchored onto 12 pseudochromosomes (2*n* = 24, Table 1). The final chromosome-level genome assembly of *C. camphora* is 680.79 Mb, with a scaffold N50 of 60.04 Mb. Our assembled genome size of *C. camphora* is very similar to the estimated genome size of 687.08 ± 13.38 Mb/1C according to flow cytometry analysis (Additional file 2: Fig. S2a, b).

**Table 1:** Comparisons among five genome studies of *Cinnamomum camphora*

**Studies**		**Shen et al. 2022 **[21]	**Sun et al. 2022 **[22]	**Jiang et al. 2022 **[23]	**Wang et al. 2022 **[24]	**The present study**
**Plant materials for genome sequencing**		NA	A mature individual which is growing naturally in Yong Chun, Quan-Zhou city	*C. camphora* var. *linaloolifera* Fujita; NO.95	Fresh leaves of *C. camphora* (Lin-type)	Fresh leaves of a 500-year-old *C. camphora* in Wuxue, Hubei
**Genome assembly**	Predicted genome size (Mb) and method	785 /17-mer analysis	719.93 /K-mer analysis	760 /flow cytometric analysis	723.12 /K-mer analysis	687.08 /flow cytometric analysis
	Genome size (Mb)	NA	737.85	755.41	706.47	680.79
	chromosome-level size (Mb)	670.29	732.69 (99.3%)	697.81 (92.38%)	703.92 (99.5%)	666.5 (97.9%)
**Quality assessment**	Contig N50 (Mb)	2.41	2.60	2.01	2.19	9.18
	Scaffold N50 (Mb)	60.19	66.26	64.34	3.17	60.04
	Busco assessment for genome sequences	95.20%	95.27% embryophyta_odb10	96.2% viridiplantae	95.2% embryophyta_odb10	99.0% embryophyta_odb10
	Busco assessment for protein sequences	90.80%	89.75% embryophyta_odb10	NA	90% embryophyta_odb10	97.6% embryophyta_odb10
	Mapping/coverage rate	NA	NA	93-95% mapping rates of RNA-Seq paired-end reads against the assembled genome	The coverage rate obtained from five BAC clones and RNA-seq unigenes was 88.58 ∼ 99.83% and 98.20%, respectively	Average DNA-Seq mapping rate was 95.64%, and average RNA-Seq mapping rates was 91.81%.
**Genome Annotation**	No. of predicted proteins	29,919	29,789	24,883	36,411	34,918
	No. of protein with functional annotation	NA	26,583 (89.24%)	24,152 (97.06%)	30,117 (82.71%)	30,076 (86.13%)
**TPS Gene identification**	No. of TPS genes	83	85	72	78	79
	No. of TPS-b genes	42	54	44	32	44

NA represents data not available

We annotated the *C. camphora* genome using Maker pipeline v2.31, incorporating ab initio predictions (Augustus v3.4.0, GeneMark-EP v4.63, and SNAP v2013-11-29), homology-based evidence (all land plant protein sequences in the OrthoDB v10.1 database), and RNA-sequencing (RNA-seq) data, resulting in 34,918 protein-coding genes. Of these genes, 7572 ones were tandemly duplicated identified by MCScanX inserted in TBtools (Additional file 1: Table S1). In addition, 56.02% of the genome was annotated to be repeat sequences, of which long terminal repeat (LTR) elements accounted for the largest proportion (Additional file 1: Table S2).

For genome quality assessment, the proteome was estimated to be at least 97.6% complete based on BUSCO assessment [28], which is higher than that of published *C. camphora* genomes (89.75% [22], 90.8% [21], and 90% [24]) (Table 1) and other sequenced plant species in Lauraceae, i.e. *C. kanehirae* (89%) [3], *Litsea cubeba* (88.4%) [1], *Persea americana* (85% and 86.3%) [4], and *Phoebe bournei* (81.1%) [5]. All the paired-end reads from Illumina sequencing were mapped against the final assembly of *C. camphora*, corresponding to 95.64% of the total mapped reads. Additionally, RNA-seq reads from different tissues and cold acclimation treatments were also mapped back to the genome assembly using HiSAT2 [29], resulting in an average of 91.81% of the total mapped RNA-seq reads. These results from the DNA or RNA read mapping and BUSCO analyses supported the completeness and high reliability of the reference *C. camphora* genome. Detailed comparisons using a series of approaches between the genome in this study and published *C. camphora* genomes also validated a high-quality reference assembly of the *C. camphora* genome we obtained (Table 1; Additional file 2: Fig. S3a-d).

### Phylogenetic affinity, ecological niche differentiation, and population dynamics between two Cinnamomum species

**Fig. 1:**
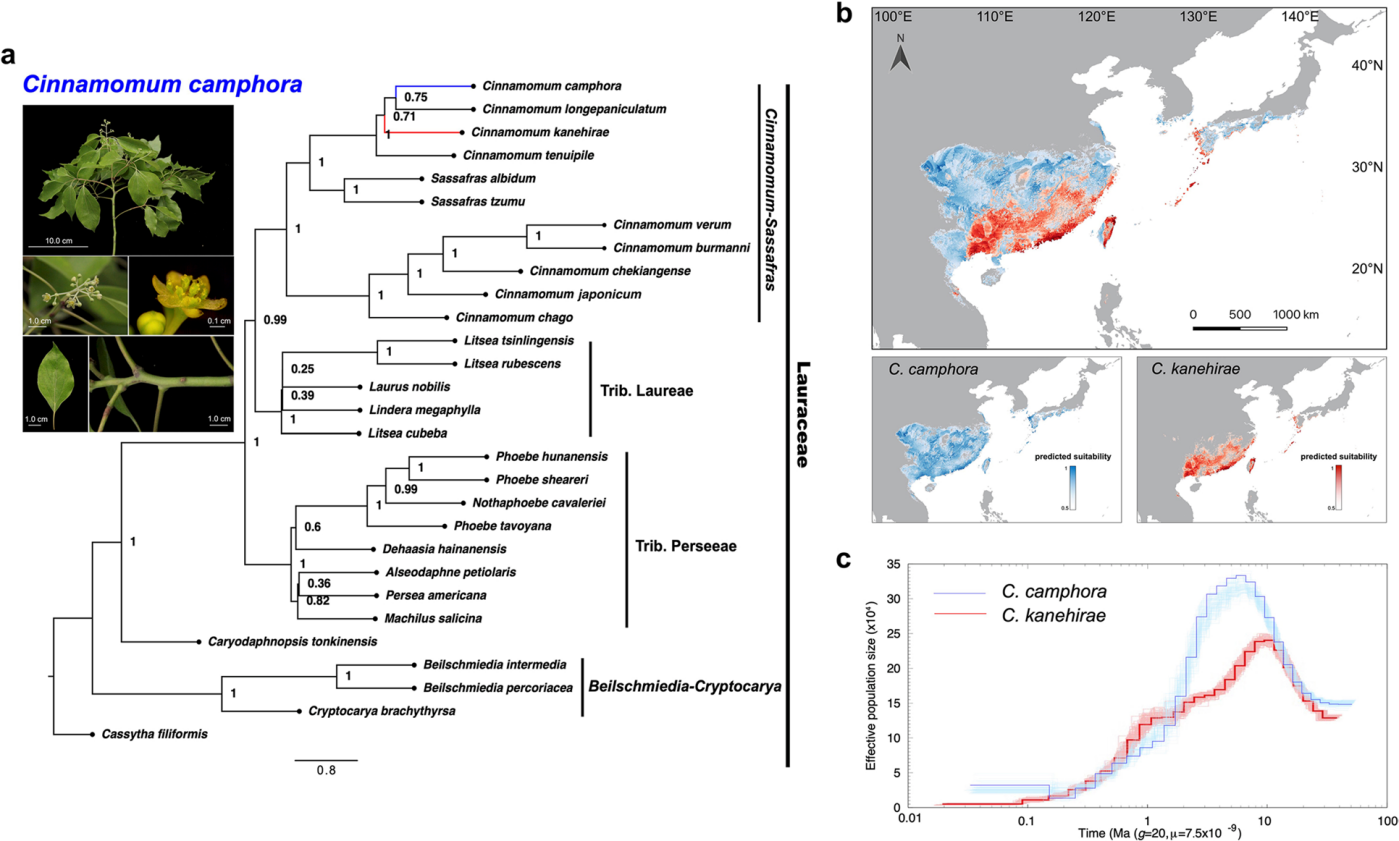
Feature overview of *Cinnamomum camphora* and its relative, *C. kanehirae*. **a** Morphological features of *C. camphora* and a phylogenetic species tree in Lauraceae. Morphological images included a branchlet, inflorescences, flower, leaf, and young stem. The species tree is based on amino acid sequences of identified single-copy OGs with a coalescent-based method from 29 Lauraceae species. **b** Inferred distribution of *C. camphora* (blue) and *C. kanehirae* (red). **c** History of effective population size predicated using the PSMC method. The blue line for *C. camphora* is based on the consensus sequences obtained from Illumina sequencing data, and the red line for *C. kanehirae* is from Chaw et al. [3]. One hundred bootstraps were performed and the margins are shown in light blue and light red for *C. camphora* and *C. kanehirae*, respectively

To validate the phylogenetic affinity between *C. camphora* and *C. kanehirae*, 296 single-copy orthologous genes (OGs) of 29 species were used to reconstruct a phylogenetic tree of Lauraceae (Additional file 1: Table S3). The phylogenetic trees inferred by the maximum likelihood (ML) method supported four robust clades: *Cinnamomum-Sassafras*, Trib. Laureae, Trib. Perseeae, and *Beilschmiedia-Cryptocarya* (Fig. 1a). Of these clades, *C. camphora* belongs to the clade of *Cinnamomum-Sassafras*, where *C. camphora* and *C. longepaniculatum* formed a small group that is sister related to *C. kanehirae*, confirming their close phylogenetic relationship. Genome collinearity analysis between these two species revealed that approximately 92% of *C. camphora* genomes matched one-to-one syntenic blocks with 91% of *C. kanehirae* genomes (Additional file 2: Fig. S4a, b). Additionally, 20,237 high-quality collinearity gene pairs were identified (Additional file 2: Fig. S4c), indicating good collinearity between these two species. Large structural variations between the two species were intrachromosomal translocations and inversions, and obvious duplications were only observed on chromosome 6 (Chr 6) (Additional file 2: Fig. S5).

Climatic niches are essential in determining the distribution of species and how they will respond to climate changes [13]; thus, we performed ecological niche models (ENMs) to explore whether the climatic niches of *C. camphora* and *C. kanehirae* have been partitioned. Our results indicated that the potential distribution of the two species is remarkably differentiated in East Asia. The suitable distribution of *C. camphora* is further north than that of *C. kanehirae*. *C. camphora* was predicted to be widely distributed from 20° N to over 30° N in China and reached 35° N in the Korean Peninsula and Japan. In contrast, the distribution hotspots of *C. kanehirae* were predicted to be coastal areas in southern China (Fig. 1b). In addition, the results of the jackknife test suggested that the BIO2 Mean Diurnal Range (mean of monthly (max. temperature – min. temperature)) contributed most to the ENMs of *C. camphora* and *C. kanehirae* (Additional file 2: Fig. S6a, b). To explore the population dynamics of *C. camphora* and *C. kanehirae*, we compared the effective population size (*N*_e_) of the two species, which suggested a larger expansion of *C. camphora* than *C. kanehirae* with an obvious peak at 6–7 million years ago (Ma). Subsequently, a continuous and rapid decline of *N*_e_ in *C. camphora* appeared from 3 Ma to around 0.15 Ma, while the decline of *N*_e_ in *C. kanehirae* was much gentler than that of *C. camphora* during this period (Fig. 1c). Nevertheless, there was a small expansion of *C. camphora* relative to the continuous decline of *N*_e_ in *C. kanehirae* over the past 0.15 Ma*.* Overall, *C. camphora* and *C. kanehirae* have highly similar genetic backgrounds but contrasting ecological niches, which lays the foundation for subsequent comparative genomics investigation.

### Comparative genomics indicated the reasons contributing to enhanced environmental adaptation

To further compare the genomes between *C. camphora* and *C. kanehirae* according to gene function, we first determined unique and shared protein domains in *C. camphora*, *C. kanehirae*, and representative species (*Amborella trichopoda*, *Arabidopsis thaliana*, and *L. chinense*). In summary, 3957 Pfams were shared by five species, 131 were specific to *C. camphora* and *C. kanehirae*, and 23 were only found in *C. camphora* (Additional file 2: Fig. S7a). There were 25 genes in Pfams specific to *C. camphora,* and these genes were significantly overrepresented in GO terms related to ‘auxin polar transport’, ‘triglyceride biosynthesis and metabolism’, and ‘circadian rhythm’ (Additional file 2: Fig. S7b). Two genes involved in the above pathways were Ccam01g03083 and Ccam01g03084, both of which are homologues of AT4G24500 [30] (*RON3*, full names of gene abbreviations listed in Additional file 1: Table S4). The coexpression networks of *RON3* from Pfams specific to *C. camphora* were further analysed (Additional file 2: Figs. S8a, b, and S9). Specifically, the stress response transcription factors NAC [31, 32], MYB [33, 34], WRKY [35–37], and HD-ZIP [38, 39], and numerous functional proteins related to ABA signalling and transportation [40–42], such as OST1, ABI1, ABCG40, and cold response genes [43–45] (*CBF5*, *ASK4*, *KCS1*, *CHY1*), were found in the coexpression network. Furthermore, two important transcription factors in circadian rhythm, i.e. BBX7 [46] and TCP15 [47], were coexpressed with *RON3*.

**Fig. 2:**
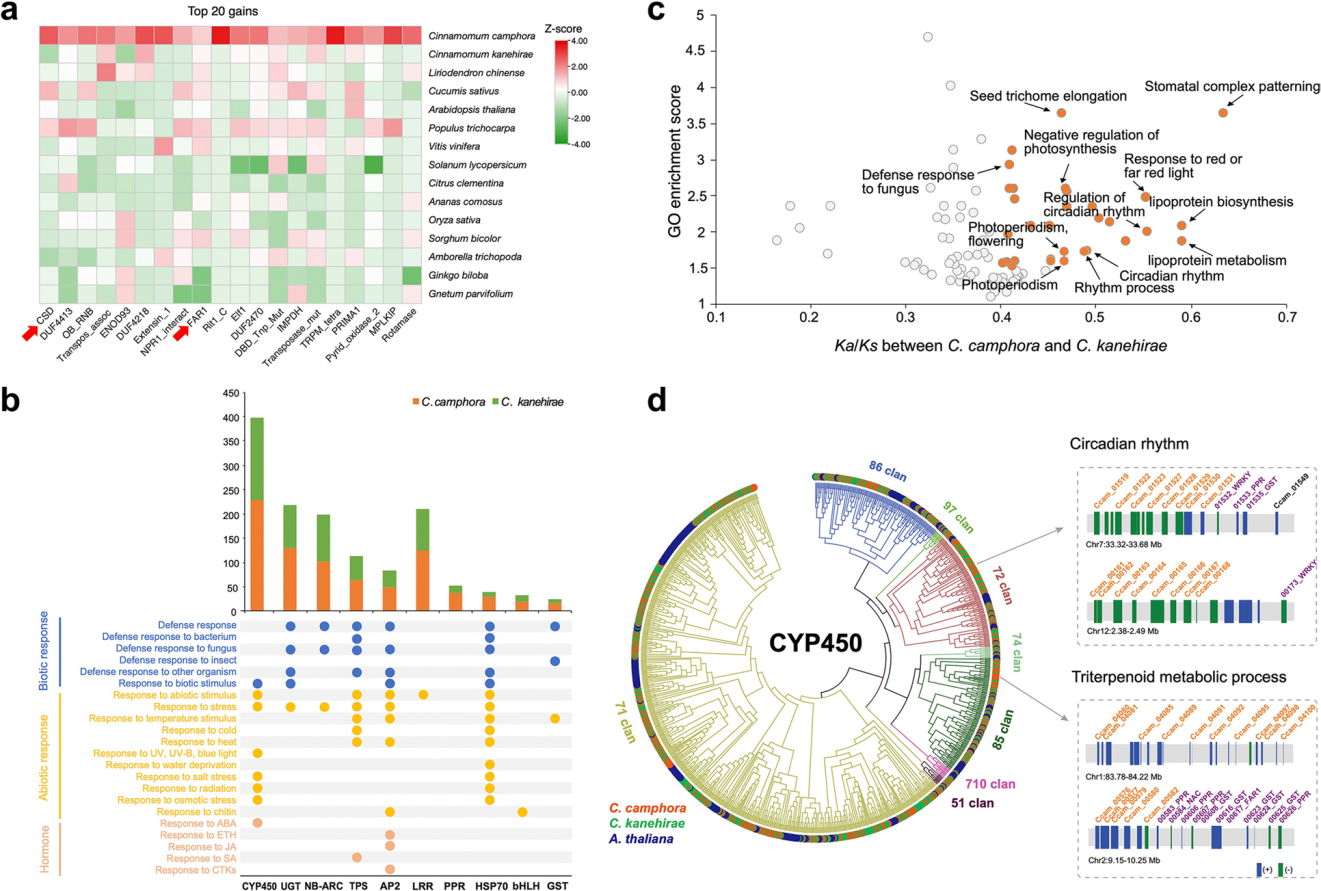
Comparative genomics analyses between *Cinnamomum camphora* and *C. kanehirae*. **a** Top 20 significant Pfam gains in *C. camphora* sorted by the ratio of domain counts between *C. camphora* and *C. kanehirae*. The cold shock domain (CSD) had the greatest gain in Pfam in *C. camphora* compared with *C. kanehirae*. For every Pfam, a *z* score was calculated for the corresponding abundance in each species. Only *z* scores greater than 1.50 or less than −1.50 are considered to be significant. **b** GO enrichment of gene pairs between *C. camphora* and *C. kanehirae* showing that genes related to circadian rhythm were positively selected. The *x*-axis represents the average *K*_*a*_*/K*_*s*_ ratios of genes in each GO term, and the *y*-axis represents the enrichment score. GO terms with an average *K*_*a*_*/K*_*s*_ ratio greater than 0.4 and enrichment score greater than 1.5 are highlighted. **c** Tandemly duplicated genes in *C. camphora* compared with *C. kanehirae*. The left panel shows enriched GO terms related to the stress response of the most tandemly duplicated gene families. The bottom and upper panels show the gene name and number of these tandem-duplicated genes*.* The dots indicate gene enrichment in corresponding GO terms. **d** Phylogenetic and tandem duplication analyses of *CYP450* genes in *C. camphora*. Branches of the phylogenetic tree are colour-coded according to *CYP450* subgroups. The right panels indicate dense tandem arrays of members from the *CYP450* 72 or 85 clan. These *CYP450* genes formed gene clusters with genes (names in purple) responding to abiotic stress on Chr 7, 12, 1, and 2

To identify protein domains and genes expanded in *C. camphora* compared with *C. kanehirae*, we analysed the significant enrichment and the reduction of protein domains (Fig. 2a). Interestingly, the cold shock domain (CSD) with 19 tandemly duplicated copies was the most enriched protein domain in *C. camphora*, more than that in the other species from gymnosperms, Amborellales, Nymphaeales, and Austrobaileyales (ANA grade), *L. chinense*, eudicots, and monocots, while only one copy was enriched in *C. kanehirae* (Additional file 2: Fig. S10)*.* CSD is the domain of cold shock protein (CSP), which has been demonstrated to play essential roles in acquiring freezing tolerance [48]. We found that the CSP gene family was expanded significantly in the analysis of orthogroup expansions (Additional file 2: Fig. S11), validating the substantial expansion of CSP in *C. camphora.* In addition, genes and protein domains responding to and positively increasing resistance to abiotic or biotic stress were expanded in *C. camphora*, such as FAR1 [49, 50], UGT [51–53], and P-loop NTPases [54] (Fig. 2b; Additional file 2: Fig. S11).

An increasing rate of nonsynonymous mutations within genes can also be a sign of adaptive divergence at the molecular level [55]. First, we analysed the functional enrichment of genes under positive selection, i.e. nonsynonymous substitution rate/ synonymous substitution rate > 1 (*K*_*a*_/*K*_*s*_ >1) between *C. camphora* and its 10 most closely related species according to the phylogenetic tree of Lauraceae (Additional file 2: Fig. S12a). ‘Plant‒pathogen interaction’ and ‘environmental adaptation’ were the most significantly (*p* < 0.05) enriched KEGG pathways for these genes under positive selection, which indicated a stronger ability of environmental adaptation of *C. camphora* compared with its relatives. Interestingly, a large number of genes from the MYB family underwent positive selection, among which two *CCA1-like* genes function in circadian rhythm, while others belong to the R2R3-MYB subfamily, with roles primarily related to flavonoid and lignin biosynthesis (Additional file 2: Fig. S12b). Then, the *K*_*a*_/*K*_*s*_ values between *C. camphora* and *C. kanehirae* for different GO categories revealed an extraordinary enrichment of elevated pairwise *K*_*a*_/*K*_*s*_ values in the circadian rhythm and defence response categories (Fig. 2b). These genes with elevated *K*_*a*_/*K*_*s*_ values were significantly enriched in GO terms, including ‘stomatal complex patterning’, ‘response to red or far-red light’, ‘regulation of circadian rhythm’, ‘circadian rhythm’, ‘rhythm process’, and ‘defence response to fungus’. These results indicated that genes of these functions were rapidly evolving under positive selection in *C. camphora* relative to *C. kanehirae*. Taken together, we speculated that CSPs and genes in the circadian rhythm pathway are pivotal in enhancing environmental adaptation, especially the cold adaptability of *C. camphora*.

### Tandemly duplicated genes improved the abiotic stress resistance

During evolution, genes in tandem repeats have been found to improve plant resilience to stress, particularly abiotic stress [55, 56]. We compared the differences in the number of tandemly duplicated genes between *C. camphora* and *C. kanehirae.* A total of 7572 tandemly duplicated genes were identified in *C. camphora,* with the largest number in CYP450, followed by UGT, NB-ARC, and TPS (Fig. 2c). In contrast, there were 5103 tandemly duplicated genes in *C. kanehirae,* and the numbers of genes in the main gene families were less than those in *C. camphora.* To understand the functional properties of these genes expanded by tandem duplication, we performed GO enrichment analyses. Many genes exhibited diverse functions in response to biotic stresses (insects, fungi, bacteria, and viruses), abiotic stresses (e.g. temperature stimulus, light, water deprivation, salt stress), and hormones (e.g. abscisic acid, ethylene, jasmonic acid). Of these, CYP450 genes with the largest gene numbers in *C. camphora* were primarily involved in abiotic stress (Fig. 2c).

We constructed a phylogenetic tree of all CYP450 members to explore their lineage-specific differences between *C. camphora* and *C. kanehirae* (Fig. 2d). Most members in this gene family of these two species were uniformly dispersed; however, divergent dense expansion of genes could be observed in some small branches. Specifically, genes from *C. camphora* were mainly abundant in non-A-type CYP450s, especially in the 72 and 85 clans, whereas those from *C. kanehirae* were enriched in clan 71, belonging to A-type CYP450. Expression pattern analyses of genes in CYP450 gene family indicated that a large number of members exhibited high expression in flower and responses to cold (Additional file 2: Fig. S13). In particularly, most CYP72As (72 clan) were upregulated under 2 h cold acclimation treatment exhibiting response to cold, while the expression of those in CYP716A (85 clan) were tissue specific. More detailed analyses of the chromosome localization of expanded CYP450 genes in *C. camphora* suggested that most members in clan 72 were densely distributed on Chr 7 and 12, with 8 copies each. Additionally, genes in clan 85 exhibited tandem repeats on Chr 1 and 2 (10 and 5 copies, respectively). Based on the phylogenetic relationship with homologues of *Arabidopsis*, CYP72A8 was one of the tandemly duplicated CYP450 genes in clan 72 and participated in the circadian rhythm pathway. In contrast, members in clan 85 were mainly CYP716A1 in the triterpenoid metabolic process. In particular, the above tandemly duplicated CYP450s were arranged together with a large number of genes related to abiotic tolerance on Chr 2, 7, and 12, such as the transcription factors WRKY and NAC and functional genes containing PPR domains [57, 58] or belonging to the GST family. Coexpression networks of a highly expressed CYP450 gene (Ccam02g00082) confirmed the coordinate relationship between CYP450 and its adjacent genes (Additional file 2: Fig. S14).

To further explore the relationships between tandem duplication and gene functions, we performed GO enrichment of all tandemly duplicated genes in *C. camphora*. In the biological process category, the most significantly enriched GO terms were secondary metabolic processes, including biosynthesis and metabolic processes of phenylpropanoids, lignin, and flavonoids (Additional file 2: Fig. S15). These pathways belong to phenylpropanoid metabolism, which is one of the most extensively investigated metabolic routes. In addition, we found that genes in expanded orthogroups were significantly enriched in almost the same GO terms as those above (Additional file 1: Table S5). These results suggested functional concordance between genes identified by tandem duplication analysis and expanded orthogroups. In the molecular function category, most enriched GO terms were related to the O-methyltransferase activity of flavonoids, such as luteolin, quercetin, and myricetin (Additional file 2: Fig. S16). We further detected the metabolites in different tissues of *C. camphora* using ultra-performance liquid chromatography quadrupole time of flight mass spectrometry (UPLC-Q-TOF/MS) analysis, and the most abundant metabolites were primarily flavonoids, such as quercetin-3-O-rhamnoside and rutin (Additional file 2: Fig. S17). These results were consistent with GO enrichment of tandemly duplicated genes, suggesting the crucial roles of flavonoids in *C. camphora*.

### The participation of the phenylpropanoid pathway in the cold response

Since temperatures, particularly low temperatures, are the primary factor leading to ecological niche differentiation between *C. camphora* and *C. kanehirae*, we performed different cold acclimation treatments (0, 2, and 12 h under 4 ℃, abbreviated as CK (control), CA2, and CA12, respectively) on leaves. To reveal the specific features under these treatments, a Venn diagram was applied to pairwise differentially expressed genes (DEGs) from three comparisons (Additional file 2: Fig. S18a). For the comparison of CK *vs*. CA2, CK *vs.* CA12, and CA2 *vs.* CA12, there were 359, 353, and 335 specific DEGs, respectively. DEGs following a short cold acclimation (control *vs*. CA2) demonstrated the role of stress and defence response, biosynthesis and metabolism of lipid, phenylpropanoid, and other secondary metabolites (Additional file 2: Fig. S18b). In contrast, carbohydrate metabolism, starch and sucrose metabolism, and energy metabolism were activated after a long cold acclimation, confirmed by DEGs specific to CK *vs.* CA12 and CA2 *vs.* CA12.

Functional enrichment of both tandemly duplicated genes and DEGs specific to short cold acclimation suggested the participation of phenylpropanoid-related pathways in the cold response of *C. camphora*. Subsequently, the expression profiles of genes involved in phenylpropanoid metabolic pathways were analysed in the tissues of leaves, stems, and flowers under different cold acclimation treatments (Additional file 2: Fig. S19). In the general phenylpropanoid pathway, all transcripts encoding phenylalanine ammonia-lyase (PAL) exhibited high expression in flowers and increased expression levels in samples with prolonged exposure under cold acclimation. In contrast, only one transcript encoding catalyses 4-hydroxylation (C4H) and three transcripts encoding 4-coumarate-CoA ligase (4CL) had relatively elevated expression levels, which presented similar patterns to those of genes encoding PAL. The two most highly expressed transcripts (Ccam07g01382 and Ccam12g00052) encoding chalcone synthase (CHS) were enriched in samples of stems, flowers, and CA12. Nevertheless, there was no obvious differential expression of transcripts encoding chalcone isomerase (CHI), flavonoid 3’-monooxygenase (F3’H) and naringenin 3-dioxygenase (F3H). The transcripts encoding flavonol synthase (FLS) were mainly upregulated in flowers and samples of CA2. For lignin biosynthesis, most transcripts encoding key enzymes were obviously upregulated in flowers, except for transcripts encoding caffeoyl shikimate esterase (CSE). In addition, transcripts encoding shikimate O-hydroxycinnamoyltransferase (HCT), CSE, and ferulate-5-hydroxylase (F5H) were distinctly increased in CA2. Overall, genes involved in phenylpropanoid metabolism were differentially expressed in leaves, stems, and flowers and exhibited obvious cold response patterns.

### Dual roles of terpene biosynthesis genes to enhance the biotic tolerance and produce aromatic compounds in *Cinnamomum camphora*

TPSs are key enzymes that regulate the production of terpenoids, which have been reported to be the main volatiles in *C. camphora* [10, 21]. Interestingly, more tandem repeats of this gene family were exhibited in *C. camphora* than *C. kanehirae*, and these genes could respond to diverse biotic and abiotic stimuli, including cold (Fig. 2c). To explore the evolutionary differences in the TPS family, we searched for candidate TPSs in *C. camphora*, *C. kanehirae*, and other flowering plants (Additional file 2: Fig. S20a, b; Additional file 1: Table S6). TPSs exhibited the most remarkable expansion in magnoliids relative to ANA grade. Briefly, *C. camphora* encodes 79 TPSs, while the largest number of TPSs was identified in *C. kanehirae* [3]. In consideration of the subfamily classification, TPSs were divided into 7 clades: TPS-a, b, c, d, e, f, and g. Of these TPSs, a large number of TPS-a and TPS-b were particularly detected in *C. camphora* and *C. kanehirae*, which directly contributes to their significant expansion of this gene family.

**Fig. 3:**
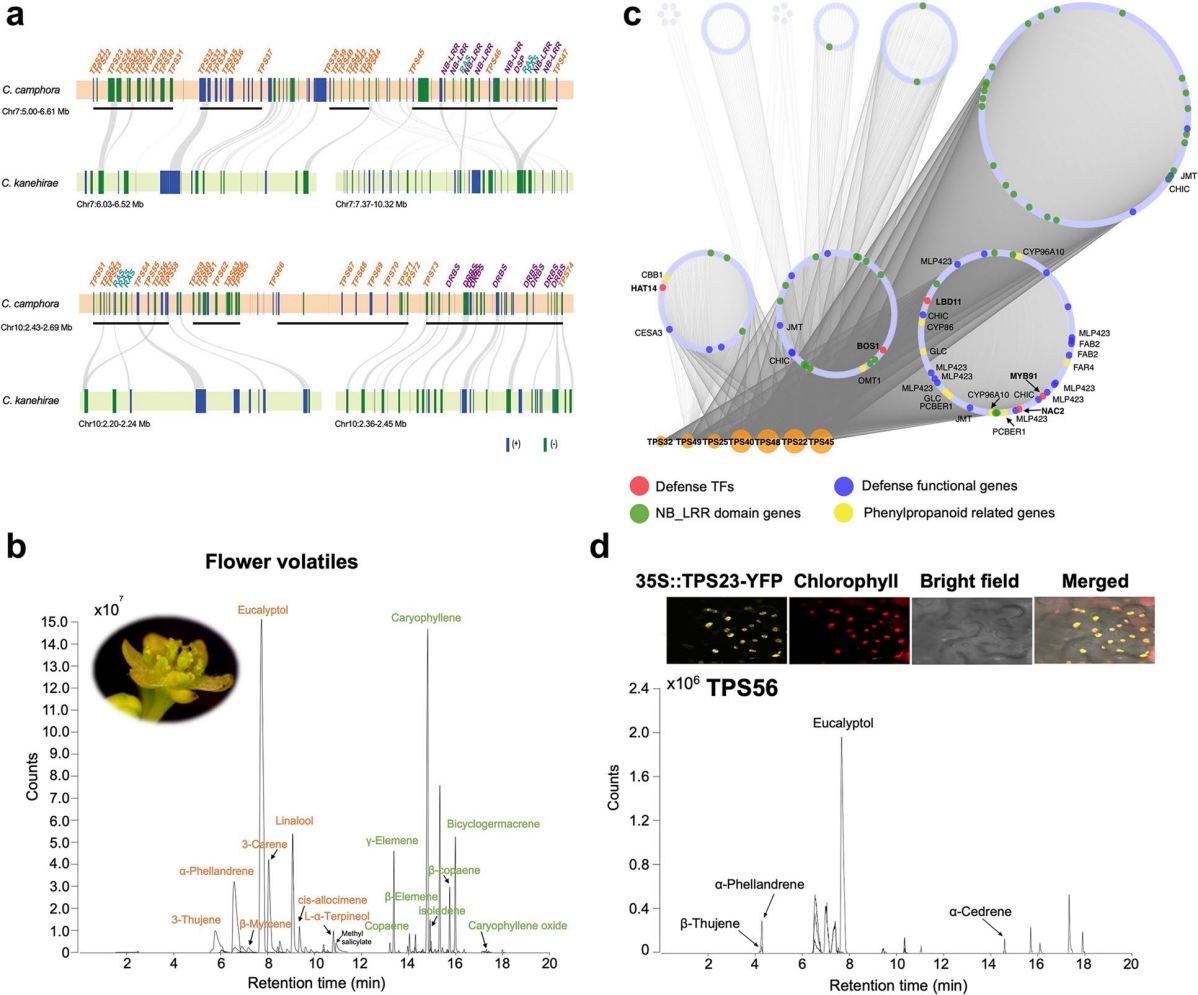
*Terpenoid biosynthesis* (*TPS*) gene characterization, synergy with defence response genes, and function validation. **a** Arrangement and chromosome location of densely tandemly arrayed *TPS* gene clusters and adjacent genes of *Cinnamomum camphora*. Black lines under genes indicate tandem duplication clusters. Intergenomic synteny blocks of these *TPS-*located regions between *C. camphora* and *C. kanehirae* are linked by grey lines. Genes with names in purple are defence responses to biotic stress, while those in blue‒green are responses to abiotic stress. DRBS, defence response to biotic stress; RAS, response to abiotic stress. **b** Coexpression networks of *TPS* genes with weight values greater than 0.5. Node size is based on degree value, and nodes are grouped according to closeness centrality analysed in Cytoscape. **c** Flower volatiles of *C. camphora* by GC‒MS analysis. Compounds shown in orange are monoterpenes, green are sesquiterpenes, and black are nonterpene compounds. **d** Subcellular localization and transient function validation of highly expressed *C. camphora TPS* genes in tobacco. Each group of upper panels shows images of the subcellular localization of TPS-YFP fusion proteins in tobacco mesophyll cells. Each bottom panel is the chromatogram of volatile compounds detected in tobacco leaves by GC‒MS analysis

To further investigate microevolutionary differences in TPSs between *C. camphora* and *C. kanehirae*, we analysed the characteristics of chromosome localization (Fig. 3a). TPSs exhibit obvious tandem repeats, especially on Chr 7 and 10 in *C. camphora*. Of these, concentrated TPSs, including *TPS21* to *TPS47*, were tandemly duplicated within the 5.00–6.61-Mb region on Chr 7. Similarly, *TPS45*, *TPS46*, and *TPS47* formed gene clusters with a large number of defence and several abiotic response genes, such as *RPS2*, *SBT3.5*, and *KCS11*. Analogous tandem repeats and gene clusters with defence or abiotic response genes were observed in TPSs of the 2.43–2.69-Mb region on Chr 10. Collinearity analysis of these regions between the two species indicated that more tandem repeats have occurred in partial regions of *C. camphora*, especially those with abundant defence or abiotic response genes. The chromosome localization variations of TPSs between these two species may lead to their distinct environmental adaptation, especially defence response.

To verify the relationship between distribution proximity and functional synergy among TPSs and these defence response genes, we examined coexpression networks of all expressed TPSs (Fig. 3b). Interestingly, TPSs with high weights between their coexpressed genes were distributed on Chr 7 and were in the blue module, which showed a significantly high correlation with flowers (Additional file 2: Fig. S21a). GO enrichment of coexpressed genes suggested their crucial roles in ‘single-organism process’, ‘regulation of hormone levels’, ‘phenylpropanoid metabolic process’, ‘interaction of host’ and those related to reproduction (Additional file 2: Fig. S21b). These coexpressed genes comprised many genes with NB_LRR domains, genes encoding defence response-related transcription factors (HAT14, BOS1, LBD11, NAC2, and MYB91) and functional proteins (CESA3, JMT, CHIC, MLP423, and FAB2), and genes in the phenylpropanoid metabolic pathway (*CBB1*, *OMT1*, *CYP96A10*, *CYP86*, *FAR4*, *GLC*, and *PCBER1*). Taken together, the distribution proximity and coexpression of TPSs and defence response genes indicated their functional synergy in improving biotic tolerance in *C. camphora*.

Other than the contribution to the enhanced biotic tolerance, TPSs led to the production of main aromatic compounds in *C. camphora*. The aromatic compounds in the flowers, leaves, and stems of *C. camphora* were determined using gas chromatography‒mass spectrometry (GC‒MS) analysis (Fig. 3c; Additional file 2: Fig. S22a, b; Additional file 1: Table S7). The compounds detected were mainly comprised of monoterpenes and sesquiterpenes and only a small amount of methyl salicylate in flowers. Notably, both the types and contents of terpenes were the most abundant in flowers. Monoterpenes, including eucalyptol, 3-thujene, α-phellandrene, β-myrcene, linalool, *cis*-allocimene, and L-α-terpineol, and sesquiterpenes of isoledene were only identified in flowers. In leaves, there are seven monoterpenes and nine sesquiterpenes, among which (+)-2-bornanone had the highest content, followed by caryophyllene. By comparison, caryophyllene is the principal constituent in stems. The types of sesquiterpenes in stems are the same as those in leaves except for the presence of caryophyllene oxide. The monoterpenes in stems contain five compounds with low contents.

Since TPSs are considered as the primary enzymes in the biosynthesis of terpenoids, further gene expression analysis in *C. camphora* was investigated, which indicated that most TPS members showed low expression (Additional file 2: Fig. S23). Interestingly, TPSs with relatively high expression are distributed on Chr 7 and 10 and are mainly abundant in flowers, suggestive of tissue-specific expression. To validate the biological function of those TPSs with high expression, we detected the subcellular localization and the expression product of three TPSs (TPS45, 56, and 79) in tobacco leaves (Fig. 3d; Additional file 2: Fig. S24a, b, c). The results clearly showed that TPS56 is distributed throughout plastids and TPS79 in the cytosol, with the main products eucalyptol and α-farnesene, respectively. In contrast, the in vivo expression of TPS45, with the highest expression in *C. camphora,* generates both monoterpenes ((+)-2-bornanone) and sesquiterpenes (copaene, α-terpiene, γ-elemene, β-selinene, δ-cadinene), corresponding to subcellular localization in both the plastid and cytosol. Altogether, these TPSs with high expression exhibited dual roles to enhance defence response and produce the main aromatic compounds in *C. camphora*.

### Genome resequencing and the potential origin of *Cinnamomum camphora*

**Fig. 4:**
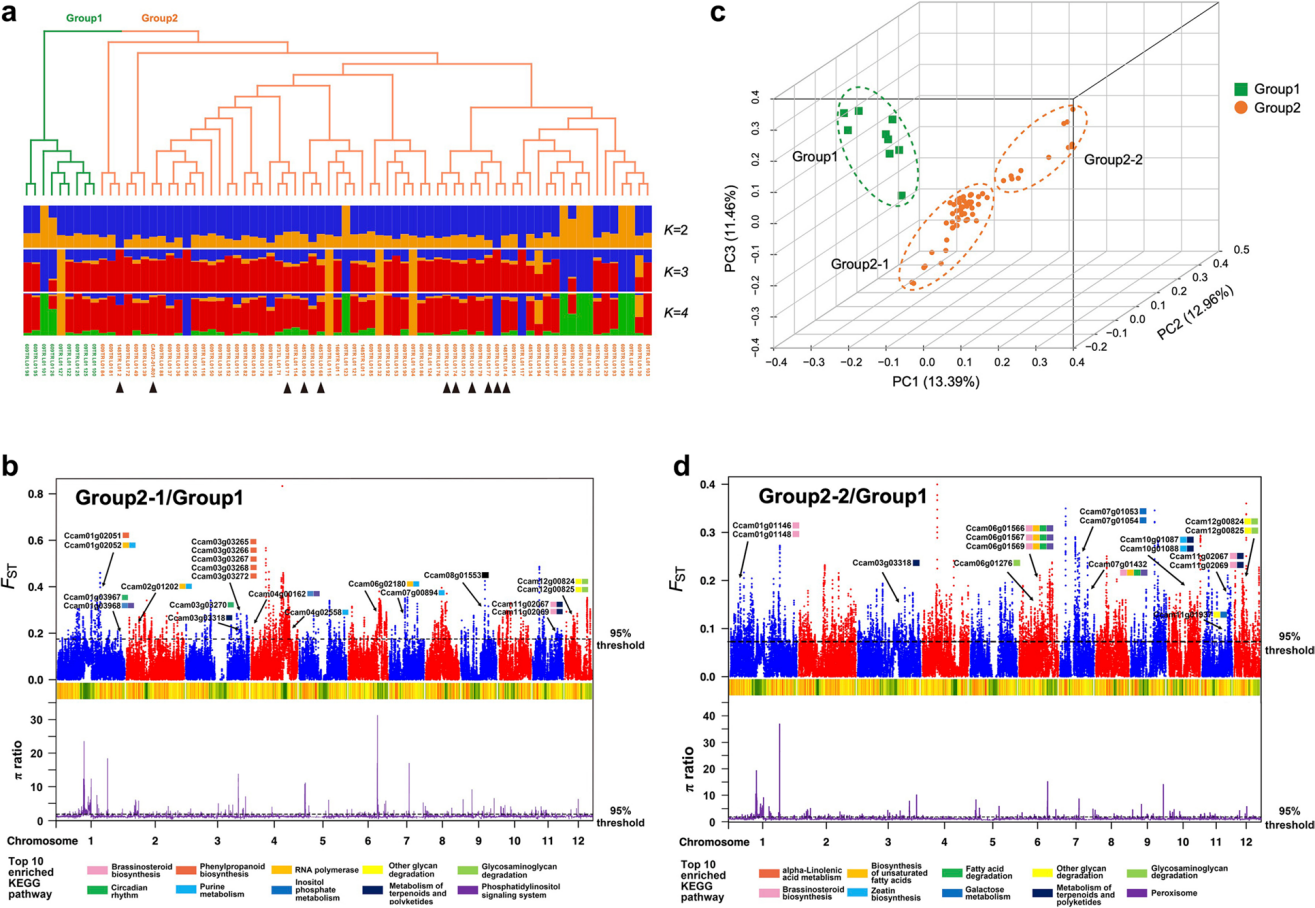
Population analyses for resequenced individuals of *Cinnamomum camphora*. **a** A neighbour-joining phylogenetic tree and population structure plots (*K* = 2, 3, 4) of all accessions (*n* = 75) estimated based on high-quality SNPs. Two populations were divided according to the phylogenetic tree and assigned as Group 1 and Group 2. Individuals of *C. camphora* greater than 100 years old are marked by black triangles. **b** Principal component (PC) analysis plots of the first three components. The fraction of the variance explained is 13.39% for PC1, 12.96% for PC2, and 11.46% for PC3. Group 2 was divided into two subpopulations based on the PC analysis. **c** The *F*_*ST*_ distribution and density of the nucleotide diversity (π) ratio between Group 1 and Group 2-1. **d** The *F*_*ST*_ distribution and density of the π ratio between Group 1 and Group 2-2. The upper Manhattan plot represents the distribution of *F*_*ST*_ in each chromosome, and the lower line plot represents the distribution of the π ratio. The top 5% of *F*_*ST*_ values and π ratios are drawn as black dashed lines. Green and yellow heatmaps indicate the density of SNPs. The combination of extremal values of *F*_*ST*_ and the π ratio together defines ‘selective sweeps’ in this study. Genes with selective sweep signals in the top 10 enriched KEGG pathways are labeled

Given its long introduction history in East Asia, wild *C. camphora* individuals are rare. To assess the phylogeny and genetic variation of cultivated *C. camphora* during cultivation and breeding, we used the accession for genome sequencing (the 500-year-old sample from Wuxue city, Hubei Province) and randomly selected 74 individuals ranging from 10 to over 700 years old in Hangzhou (Additional file 1: Table S8). A total of 621.68 Gb clean data for whole-genome resequencing were generated, yielding an average coverage of 13 × per accession. The rich dataset allowed us to identify 11.7 million SNPs to explore *C. camphora*’s genetic changes and elucidate its breeding and cultivation history. Both the neighbour-joining (NJ) tree (Fig. 4a) and the principal component analysis (PCA) (Fig. 4b) indicated that 75 accessions formed two distinct groups. Interestingly, the accessions in Group 1 were all derived from Hangzhou and were less than 60 years old. Conversely, all accessions over 100 years old belonged to Group 2, which also included the 500-year-old accession from Hubei Province, implying the potential single origin of these cultivated *C. camphora*. To verify the findings, whole-genome sequencing data of *C. kanehirae* was applied as the outgroup to identify variants and reconstruct the phylogeny of 75 *C. camphora* accessions (Additional file 2: Fig. S25). Eight out of nine accessions from Group 1 clustered together, forming sister clades with the cluster comprising one accession in Group 1 and three in Group 2, confirming the original groups and single cultivation origin of *C. camphora*; this was validated by genetic structure analyses (Fig. 4a) since there was no best ADMIXTURE solution to assign these individuals. Besides, accessions over 100 years old were dispersed in different branches, suggesting no correlation between tree ages and divergence time.

Rather than the cultivation origin, the *C. camphora* geographic origin also attracts attentions. Due to the limited sample collection sites of these resequenced accessions, available *C. camphora* genome and transcriptomes (*C. kanehirae* and *C. longepaniculatum* as the outgroups) from different sites were used to construct a species tree based on single-copy OGs (Additional file 2: Fig. S26). The results showed that the phylogeny of *C. camphora* accessions seems to have relationship between their locations except for one accession from Guangdong Province (GD, Ccam_SRR15170716). Also, accessions from Guangxi Province diverged earlier than the other *C. camphora* accessions, indicating Guangxi as its potential geographic origin. Despite the efforts to explore the origin of *C. camphora* in this study, the results were limited to the present available samples.

### Genetic changes of popular *Cinnamomum camphora* accessions in subtropical urban landscapes

The obviously larger number of accessions in Group 2 compared with Group 1 strongly suggested that those in Group 2 were more popular during *C. camphora* breeding and cultivation. To further examine the divergence between these two groups and investigate genetic changes with selection sweep signals, we estimated their population-differentiation statistics (*F*_*ST*_) and nucleotide diversity (*π*) ratios. Due to the large number and divergence of accessions in Group 2, two subgroups were divided based on PCA (Fig. 4b). We obtained similar *π* ratios of 1.204 and 1.227 for Group 2-1/Group 1 and Group 2-2/Group 1, respectively. However, a higher *F*_*ST*_ (0.073) was generated between Group 1 and Group 2-1, in contrast to a lower *F*_*ST*_ (0.044) between Group 1 and Group 2-2, indicating that a more distinct divergence occurred between Group 1 and Group 2-1. Then, genomic regions that were subject to selection as inferred from both extreme high (top 5%) *F*_*ST*_ and *π* ratios between groups were detected. For Group 2-1/Group 1, the cut-offs of *F*_*ST*_ and the *π* ratio were 0.175 and 1.866, respectively (Fig. 4c). In contrast, the cut-off of *F*_*ST*_ (0.105) decreased significantly in Group 2-2/Group 1 (Fig. 4d), which was in accordance with the lower divergence between these two groups. Moreover, the cut-off of the *π* ratio for Group 2-2/Group 1 was 1.802.

To understand the function of genes with selection sweep signals, the top 10 enriched KEGG pathways were visualized (Fig. 4c, d). ‘Brassinosteroid biosynthesis’, ‘phenylpropanoid biosynthesis’, ‘circadian rhythm’, and ‘metabolism of terpenoids and polyketides’, etc., were represented for Group 2-1/Group 1. Specifically, five of six genes in ‘phenylpropanoid biosynthesis’ were tandemly duplicated on Chr 3. In addition, *COP1* (Ccam01g03967) and *LHY1* (Ccam03g03270) in ‘circadian rhythm’ were under selection. For Group 2-2/Group 1, ‘alpha-linolenic acid metabolism’, ‘biosynthesis of unsaturated fatty acids’, ‘metabolism of terpenoids and polyketides’, and two pathways involved in phytohormone biosynthesis were enriched. Among them, genes in ‘alpha-linolenic acid metabolism’ were *JMT* (Ccam01g01146, Ccam01g01148), *ACX1* (Ccam06g01566, Ccam06g01567, Ccam06g01569), and *KAT2* (Ccam07g01432), which lead to jasmonic acid biosynthesis and are involved in the defence response. Taken together, our analyses confirmed that more popular *C. camphora* accessions underlying breeding and cultivation exhibited selection sweep signals in enhanced cold and defence responses.

### Genetic diversity and selection analysis of *Cinnamomum camphora*

**Fig. 5:**
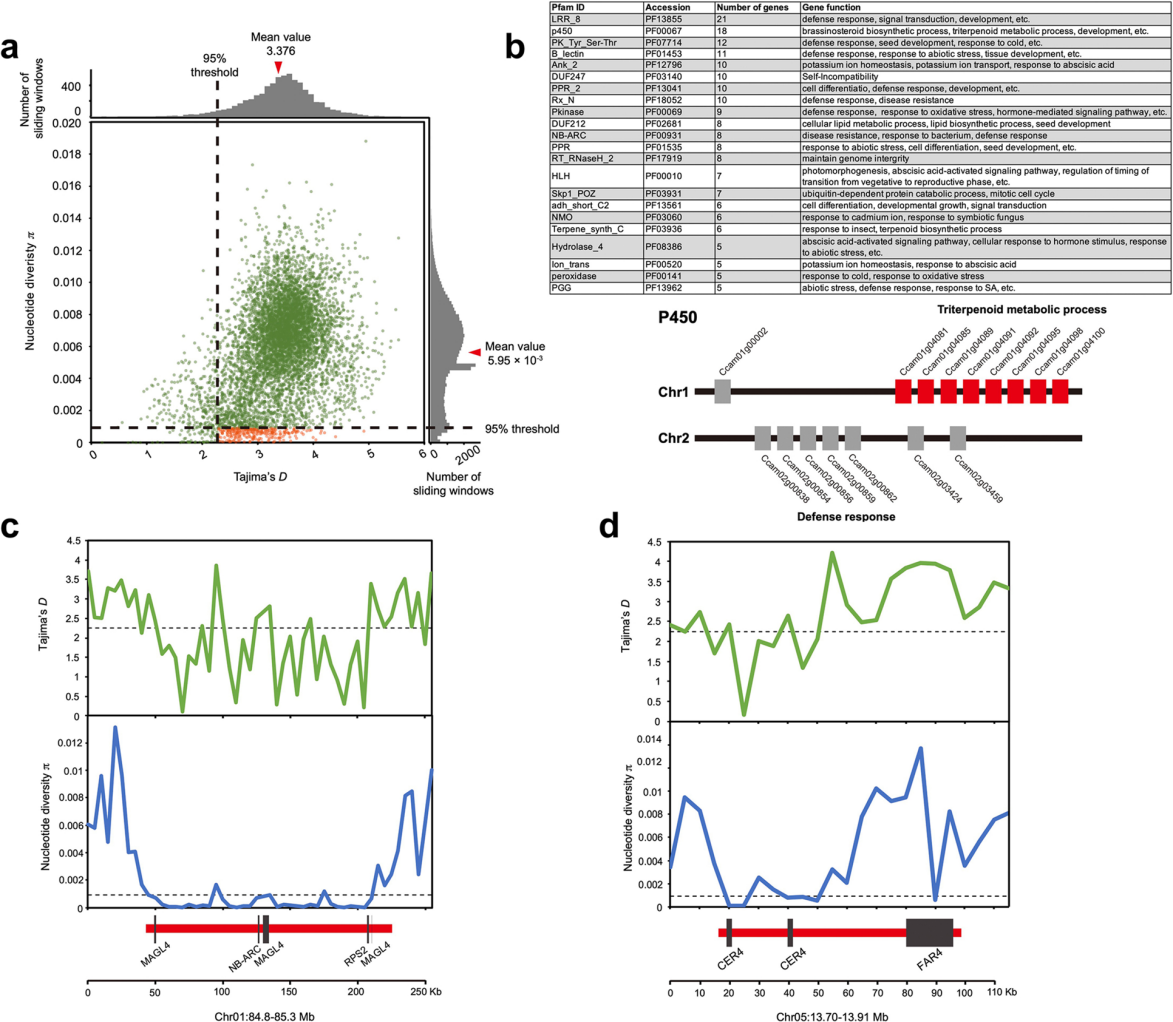
Genomic regions with balancing selection in *Cinnamomum camphora*. **a** The distribution of Tajima’s *D* in 100-kb sliding windows across the genome and nucleotide diversity (π) in 100-kb sliding windows with a 10-kb step. Average values are marked by red triangles. Orange dots represent windows fulfilling the selected requirement (corresponding to Tajima’s *D*>2.249 and *π*<0.910 × 10^−3^). **b** Gene number and function of the main Pfams with balancing selection. The upper panel shows the Pfam ID, accession, corresponding gene number, and function. The lower panel is an example of the chromosome location of tandem-duplicated genes with balancing selection and belonging to P450. **c** Genes with balancing selection signals that were significantly (*p* value < 0.05) enriched in the environmental adaptation pathway. **d** Genes with balancing selection signals that were significantly (*p* value < 0.05) enriched in the cutin, suberin, and wax biosynthesis pathways. Tajima’s *D* and *π* values are plotted using a 5-kb sliding window. Black dashed lines represent the 95% significance threshold for the whole genome of corresponding values. Genes are shown at the bottom

According to the high-density SNP data, the π of the *C. camphora* population was estimated to be 5.95 × 10^−3^ (Fig. 5a). In this study, almost all genomic regions in *C. camphora* exhibited positive Tajima’s *D* values with an average value of 3.376, suggesting that those regions were under balancing selection to maintain genetic variation. Since balancing selection plays crucial roles in adaptative evolution in diverse organisms, we paid attention to genomic regions that have been subject to significant balancing selection (Tajima’s *D* >2.249) and extremely decreased nucleotide diversity (π <0.910 × 10^−3^). A total of 1384 protein-coding genes (Additional file 1: Table S9) were harboured in these regions, which are expected to represent targets of strong balancing selection. Among them, the main enriched Pfams functioned in defence response, plant development, and response to abiotic stress, such as cold (Fig. 5b). We closely investigated genes in P450 and found that 16 of 18 genes were tandemly duplicated on Chr 1 and Chr 2. Moreover, eight genes on Chr 1 were involved in the terpenoid metabolic process, which also showed clade expansion (in contrast to *C. kanehirae*) (Fig. 2d), and five genes on Chr 2 were related to the defence response.

KEGG enrichment analysis was also conducted for these 1384 genes. The most enriched pathways were ‘cutin, suberin and wax biosynthesis’, ‘preoxisome’, and ‘plant‒pathogen interaction’, suggesting pivotal roles of these genes in biotic and abiotic tolerance as well. As examples, two genomic regions on Chr 1 and Chr 5 showed selection signals with decreased genetic diversity and a significantly higher Tajima’s *D* (Fig. 5c, d). On Chr 1, three homologues of *Arabidopsis MAGL4* involved in the ‘glycerolipid metabolism’ pathway were associated with diverse abiotic stresses and hormone responses. The other two genes with NB-ARC domains, one of which is the homologue of *Arabidopsis RPS2*, are key components of resistance genes involved in the defence response. Both *CER4* and *FAR4* on Chr 5 encode alcohol-forming fatty acyl-CoA reductases and are involved in cuticular wax and suberin biosynthesis processes. Overall, most genes subject to balancing selection were involved in environmental adaptation, while only a few genes were associated with economically significant traits.

Based on a pan-genome/transcriptome analysis, the gene function of private and shared gene family between three *C. camphora* genomes were analysed in details (Additional file 2: Fig. S27a, b). To facilitate the comparison, the genomes of our study and three published ones [22–24] were assigned as Ccam_HB, Ccam_GX, Ccam_JX, and Ccam_FJ, respectively. KEGG enrichment of genes in these families indicated that basic vital activities were significantly enriched in private genes of Ccam_GX, while pathways related to enhanced biotic and abiotic responses, such as ‘environmental adaptation’, ‘plant-pathogen interaction’, ‘phenylpropanoid biosynthesis’, and ‘alpha-linolenic acid metabolism’, were significantly enriched in private genes of Ccam_JX and Ccam_HB (Additional file 2: Fig. S27c). Furthermore, the shared genes of three genomes were enriched in pathways of ‘carbohydrate metabolism’, ‘carbon fixation in photosynthetic organisms’ and ‘terpenoid backbone biosynthesis’, validating the common ecological and economic importance of this species (Additional file 2: Fig. S27d). Combined with the relatively earlier divergence of Ccam_GX than Ccam_JX and Ccam_HB (Additional file 2: Fig. S26), it suggested the genes enhanced biotic and abiotic responses might subject to artificial selection during cultivation and breeding. The *K*_*a*_*/K*_*s*_ values of private genes in each accession are significantly greater than those of shared ones (Additional file 2: Fig. S27e), suggesting more stronger selection occurring in these private genes than shared ones, and these private genes might contribute to the adaptive evolution of *C. camphora*. Interestingly, these findings also validated the importance of genes in ‘environmental adaptation’, ‘plant-pathogen interaction’, ‘phenylpropanoid biosynthesis’ and ‘alpha-linolenic acid metabolism’ pathways contribute to enhanced abiotic and biotic responses and lead to the dominance of *C. camphora* in the subtropical urban landscapes.

## Discussion

Currently, there have been several *C. camphora* reference genomes published due to its significant evolutionary and economic value [21–24]. However, the chromosome-scale reference genome reported in this study improves the quantity and quality of genome information compared with these published assemblies and enables more sophisticated biological studies regarding *C. camphora*. Also, the valuable resequencing data provides essential information on the population structure, genetic variation, and diversification. On the other hand, these *C. camphora* genome papers primarily focused on the biosynthesis of aromatic compounds and genetic relationship in *Cinnamomum*. The reasons for the widespread of evergreen broadleaved trees are always fascinating and have not been explored. Unlike other species in *Cinnamomum,* which are restricted to tropical regions, *C. camphora* has a broad distribution from the tropics to the transition zone between subtropical and temperate regions. Therefore, the availability of the high-quality *C. camphora* genome and resequencing data facilitate in-depth comparative genomic and population diversity analyses to elucidate the evolution and mechanisms of its dominance in subtropical urban landscapes.

**Fig. 6:**
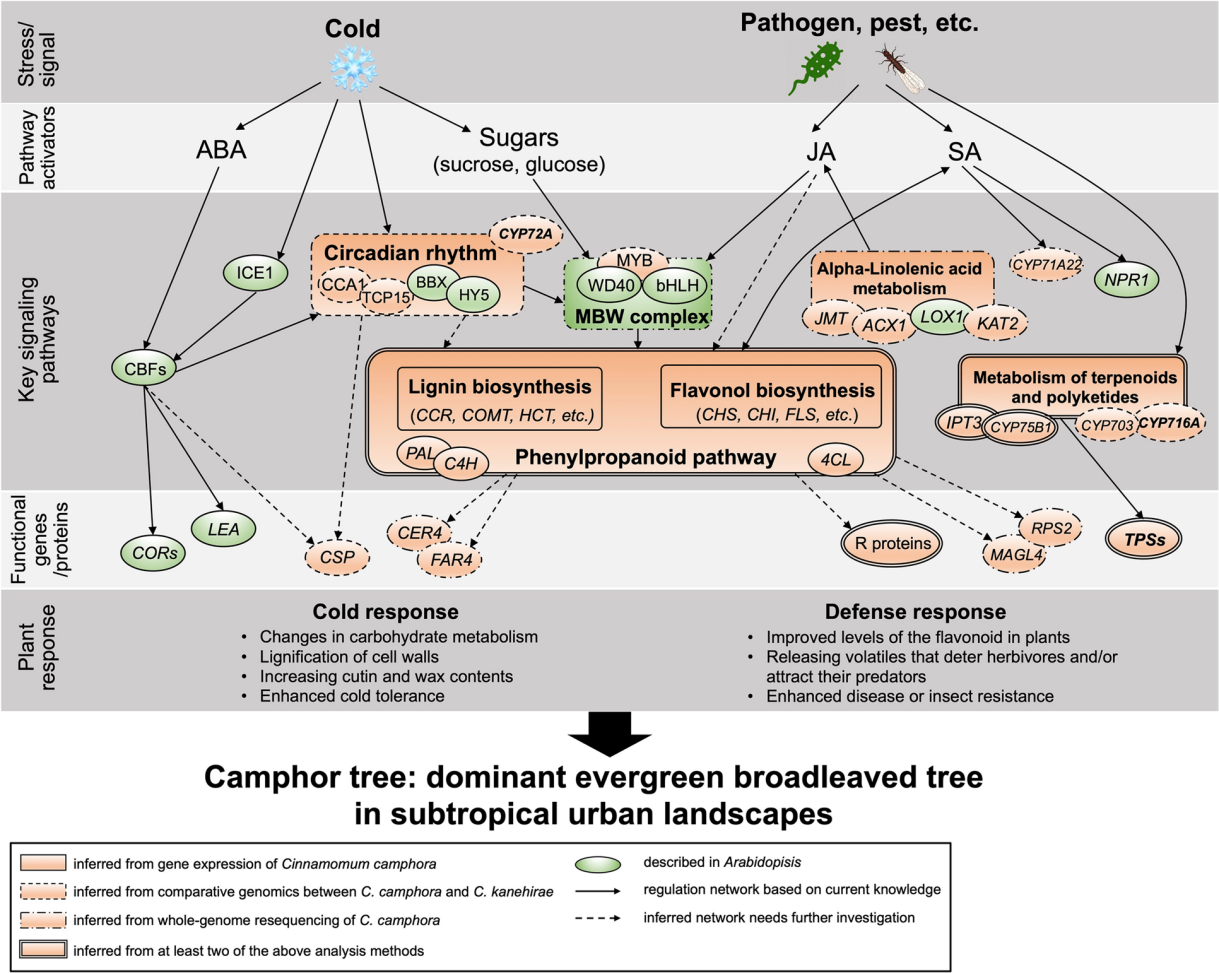
A proposed model to elucidate the reason for the dominance of *Cinnamomum camphora* in subtropical urban landscapes. Central components in the network are shown for *Arabidopsis* (light green) and *C. camphora* in this study (orange), highlighting the differences between annual herbs and evergreen broadleaved trees

To answer the above biological question, we conducted analyses from multiple levels for the first time. Firstly, *C. camphor*a and *C. kanehirae* were rendered as a fantastic system for comparative studies due to their close relationship and distinct distributions. The jackknife test result suggested low temperatures were the primary factor contributing their ecological niche differentiation, which could be confirmed by lines of evidence that the cold adaptability of *C. camphora* was enhanced relative to *C. kanehirae *(Fig. 6). Specifically, *RON3* and *BBX7* in the circadian rhythm pathway were under positive selection, while *CSPs* and those associated with phenylpropanoid metabolism expanded in *C. camphora*. A similar novel mechanism found the overexpression of *BBX7* could improve *Arabidopsis* cold tolerance independent of *CBFs* or their target *COR* genes (The CRY2–COP1–HY5–BBX7/8 module regulates blue light-dependent cold acclimation in *Arabidopsis*). Also, genes involved in circadian rhythm and phenylpropanoid-related processes of lignin and flavonoid biosynthesis have been proven to improve the cold response in evergreen trees or shrubs [59–61]. These results manifested circadian rhythm and phenylpropanoid-related processes might be functionally conservative to enhance cold tolerance in evergreens. Additionally, DEGs of *C. camphora* leaves under different cold acclimation treatments and metabolomic analysis also confirmed the significant roles of phenylpropanoid metabolism in the enhanced cold response of *C. camphora*.

Other than cold adaptability that is beneficial to the widespread distribution of *C. camphora* in subtropical regions or even higher latitudes, comparative genomics analyses indicated a significant expansion of genes associated with biotic responses via tandem duplication, such as CYP450, UGT, NB-ARC, and TPS. As reported, tandem duplication is an important type of gene expansion for adaptive evolution to changing environments [26, 62–64]. Interestingly, following gene expansion via tandem duplication, *TPS* genes formed clusters and coordinated expression with defence and biotic response genes in *C. camphora*. These metabolic gene clusters have a crucial role in facilitating rapid stress response, co-regulation of genes, and co-functioning, making them significant for plant adaptation to the environment and evolution. Moreover, TPSs encode terpene synthases to produce diverse terpenoids [1, 56], which are the primary volatiles released from almost all tissues and the main constituents of essential oils in *C. camphora* (Genome-wide analyzation and functional characterization on the TPS family provide insight into the biosynthesis of mono-terpenes in the camphor tree). Our study verified that *C. camphora* TPS56 localized in plastids, which belongs to the TPS-a group and catalyses the production of eucalyptol verified in vivo, which is the most abundant terpenoids in *C. camphora*. The product profile was similar to that of *CcTPS16* according to an in vitro assay of another *C. camphora* genome paper [24]. These results could be applied to the biosynthesis of natural terpenoids in *C. camphora*, which are precious medical material and rare natural resource known as the soft gold [65]. In addition, although obvious expansion of the TPS gene family was found in both species, tandem duplication of TPS with high expression mainly occurred in *C. camphora*, suggesting their functional divergence. Therefore, we proposed that dual roles of *TPSs* gained by tandem duplication, gene cluster formation, and gene regulation divergence may facilitate the defence response and aromatic compound biosynthesis in *C. camphora*.

Much work has been performed on annual crops to study the genetic basis of domestication and diversification in the last decade; however, little attention has been paid to perennial crops, especially trees [66]. We report a comprehensive catalogue of genome-wide genetic resources in *C. camphora* for the first time. The resequencing data here provide abundant information on genetic structure, diversification, and selection analysis during breeding and cultivation and offer candidate genes for enhanced environmental adaptation. In contrast to perennial fruit species or ornamental trees [64, 67], the breeding of *C. camphora* is much lagged. This phenomenon could be confirmed by the genetic structure results, which illustrated the possible single origin of all 75 *C. camphora* accessions and the high consistency of their genetic backgrounds. According to the high-density SNP data, lower genetic diversity was identified in *C. camphora* than in perennial woods, such as lychee [60], oak [26], and palm [66], while higher genetic diversity was identified relative to woods with abundant cultivars, such as mei [67] and peach [64], indicating the possible low domestication level of *C. camphora*. Besides, Tajima’s *D* was used to evaluate whether the observed nucleotide diversities showed evidence of deviation from selective neutrality. Some regions were significantly above zero, indicating the existence of balancing selection or a strong bottleneck [68]. In this study, almost all genomic regions were under balancing selection to maintain genetic variation. Furthermore, the positive Tajima’s *D* values were consistent with a historical bottleneck reflected by decreased *N*_*e*_ in *C. camphora* (Fig. 1c).

The geographic origin and how natural and artificial selection collaboratively shaped the genome architecture and gene makeup of *C. camphora* during long-term evolution and quality improvement are interesting biological question [69]; however, *C. camphora* has a long cultivation history and its wild ancestral populations have never been identified. Besides, there were no commercial varieties/cultivars with clear breeding records. These make it difficult to explore the origin of *C. camphora*. We took full advantage of both resequencing data and available protein sequences to try to explore the origin of *C. camphora*. The species tree based on single-copy OGs of samples from different sites indicated Guangxi was the potential geographic origin of *C. campho*r*a* according to the limited samples (Additional file 2: Fig. S26)*.* As for the cultivation origin, there was no correlation between tree ages and divergence time (Additional file 2: Fig. S25), thus group 2 including all accessions over 100 years old was not more ancient population. The obtained result was not solid enough due to the limited sample numbers and collect sites.

Although we are unable to establish a clear understanding of the domestication history of *C. camphora* currently, the two distinct groups were revealed by the phylogenetic tree and PCA. Our results indicated Group 2 was more popular in landscaping uses, implying that those in Group 2 may have more desirable traits for the dominance of *C. camphora*. Intriguingly, only a few genes subjected to selection between the two groups may be associated with specific economically significant traits, such as *CYP75B1* and *CYP703* associated with terpenoid biosynthesis [70], which contributes to the main constituents of essential oils, and *OMT* associated with lignin biosynthesis, leading to wood formation [71]. These genes were also correlated with enhanced environmental adaptation [72]. In addition, most genes with selection sweep signals between these two groups were enriched in pathways involved in stress responses. Overall, consistent with the possible low level of domestication, our analyses confirm that genetic changes in *C. camphora* are not as marked as those for most other crops to improve economic values, while enhancing environmental adaptation is the primary force driving *C. camphora* breeding and dominance.

## Conclusions

In summary, the present comparative genome and population genomic analyses provide insights into the evolutionary adaptation in subtropical regions, genetic diversity, and genetic basis of important agronomic traits in *C. camphora*. The results emphasized the significance of circadian rhythms and phenylpropanoid metabolism for the enhanced cold response in *C. camphora* compared with *C. kanehirae*. Tandem duplication of key genes in the biosynthesis pathway of aromatic compounds not only led to gene evolution but also to the formation of gene clusters with stress response genes that contribute to an improved defence response in *C. camphora*. Additionally, whole-genome resequencing analyses confirmed the crucial roles of the above pathways and revealed candidate genes under selection in more popular *C. camphora* accessions during breeding and cultivation. In conclusion, the high-quality reference genome and population genomic resources developed in this study decipher the dominance of *C. camphora* in subtropical urban landscapes and are of great value for future biological studies and breeding in *Cinnamomum* or evergreen tree species.

## Methods

### Plant material and genome sequencing

Fresh leaves, young stems, and flowers were collected from a 500-year-old *C. camphora* in Wuxue (115.56 E, 29.84 N), Hubei Province, China. High-quality genomic DNA was extracted from young leaves using a DNeasy Plant Mini Kit (Qiagen, Germany). The concentration and purity of the extracted DNA were assessed using a Nanodrop 2000 spectrophotometer (Thermo, MA, USA) and Qubit 3.0 (Thermo, CA, USA). The integrity of the DNA was determined using pulsed-field electrophoresis with a 0.8% agarose gel.

For genome sequencing, approximately 20-kb SMRTbell libraries were constructed for long-read sequencing on the PacBio Sequel platform at the Biomarker Technologies Corporation, Beijing. Additionally, short-read libraries were constructed and sequenced on an Illumina X-ten platform (Illumina, CA, USA) with 150-bp paired-end reads. After quality control, 111.06 Gb of PacBio single-molecule long reads and 49.59 Gb Illumina paired-end short reads were generated and used for assembly evaluation and genome correction. RaGOO [27] was used to anchor contigs and scaffolds to the *C. kanehirae* reference chromosome. A Hi-C library from young leaves was constructed by the Illumina Nova platform to verify the results of chromosome allocation.

### Genome assembly, annotation, and quality assessment

Canu v2.0 was used to generate an initial assembly, which was processed by purge_dups v1.0.1 and polished using NextPolish v1.3.1. RaGOO was used to anchor the scaffolds to the *C. kanehirae* reference chromosomes as *C. camphora* pseudochromosomes confirmed by Hi-C paired-end reads. Maker pipeline v2.31 were applied for genome annotation. Besides, RepeatModeler [73] and RepeatScout were used to build a de novo repeat library of the genome. Then RepeatMasker [74] (v.4.05) was used to identify and group repetitive elements.

Two strategies were applied to confirm the assembly quality of the *C. camphora* genome. The embryophyta_odb10 dataset (busco.ezlab.org) was employed to evaluate genome completeness using BUSCO [28] (v.3.0.2). All the paired-end reads from Illumina sequencing were mapped against the assembled genome using BWA-MEM [75] (v.0.7.10), and RNA-seq reads from different tissues and treatments were also mapped back to the genome assembly using HiSAT2 [29] (v.2.2.0) to assess the accuracy of the genome.

### Inference of the demographic history

We inferred the demographic history of *C. camphora* with the consensus sequences obtained from Illumina sequencing data by applying the pairwise sequentially Markovian coalescence (PSMC) model [76]. This method reconstructs the history of changes in *N*_e_ over time using the distribution of the most recent common ancestor between two alleles in an individual. The data of *C. kanehirae* was downloaded from NCBI under the BioProject accession number PRJNA477266. All of the parameters used for the PSMC program were the default parameters (-N25 -t15 -r5 -p ‘4+25*2+4+6’) with the exception of -μ 7.5 × 10^−9^ and -g 20 following the study of the *C. kanehirae* genome [3].

### Genome size estimation

The genome size of *C. camphora* was assessed by flow cytometry analysis. Genomic nuclei from 50 mg of fresh leaves were treated and incubated with the DNA fluorochrome propidium iodide (PI). Samples with three biological replicates were analysed with a BD FacsCalibur (USA) flow cytometer equipped with an argon-ion laser at Kunming Institute of Botany, Chinese Academy of Sciences, following the method of Wang et al. [77]. A total of 10,000 nuclei were analysed for each sample with constant parameters, and an average coefficient of variation (CV) <5% for G1 peaks was considered reliable. *Solanum lycopersicum* was selected as the internal standard with an estimated genome size of 900 Mb. The genome size of *C. camphora* was estimated based on the ratio of the 2C peaks between samples and the internal standard.

### Identification of single-copy orthologous genes

To identify single-copy OGs for phylogenetic analyses, we obtained transcriptomic data of 27 species and the annotated genome of *C. kanehirae* from NCBI. The data source of all 29 species used for phylogenetic analyses were summarized in Additional file 1: Table S3. Raw paired-end reads of 150 bp were processed using SOAPnuke [78] (v.1.5.2) to remove adapters and low-quality sequences. Transcripts were de novo assembled using Trinity [79] (v.2.11.0) with default parameters. After discarding redundant transcripts using CD-HIT [80] (v. 4.8.1) with a sequence identity threshold of 0.99, the longest transcript was retained. Then, coding sequences (CDSs) were predicted from the longest transcript using TransDecoder [81] (v.5.5.0) with default parameters, and the CDS encoding the longest peptide was retained for the search of OGs. Finally, we employed OrthoFinder [82] (v. 2.5.2) to search OGs from CDS across 29 species. Finally, we identified 296 single-copy OGs with at least 50% species coverage.

### Reconstruction of Lauraceae phylogeny

To determine the evolutionary affinity between *C. camphora* and *C. kanehirae*, we reconstructed the phylogeny of 29 Lauraceae species based on amino acid sequences of identified single-copy OGs with a coalescent-based method. First, amino acid sequences were aligned using MAFFT [83] (v.7.487) with default settings and trimmed using trimAL [84] (v.1.4.1) with a heuristic method (-automated1), which chooses the best gap-trimming method. Then, the ML tree of each alignment was reconstructed using RAxML-NG [85] (v.1.0.2) with a one-step ML search and bootstrap analysis (--all). The substitution model of each OG was specified with a prior run (--parse). *Cassytha filiformis* was designated as the outgroup following Chen et al. [1]. Nodes with low bootstrap support (< 10%) were collapsed using the Newick Utilities [86] for each gene tree to improve the accuracy of species tree inference [87]. Finally, the species tree was summarized using ASTRAL-III [87].

### Ecological niche modelling

To determine whether climatic niches have been partitioned between *C. camphora* and *C. kanehirae,* we generated ENMs for these two species. First, we compiled their occurrence records with accurate geographical coordinates from GBIF (https://doi.org/10.15468/dl.uveqts). Then, records with a pair-distance <5 km were excluded from raw GBIF data using the *thin* function in the R package spThin [88] to reduce the sampling bias in ENMs and to fit the resolution of bioclimatic layers. Next, we predicted suitable distribution areas for these two species based on 19 present (1970–2000) bioclimatic layers with 2.5 arc-min resolution (~5 km at the equator) (available from the WorldClim database [89] and their present occurrence data using the Maxent software [90, 91] (v.3.4.4). For each model, 10,000 random points were used to extract bioclimatic data for the environmental background. Simulations were run for 10 bootstrap replicates using random subsets (70% from the complete dataset) to train the ENMs, and testing was performed with the remaining 30%. Meanwhile, we employed the jackknife test to obtain alternate estimates of which variables are the most important in ENMs.

### Synteny, positive selection, and structural variation analyses

The longest protein sequences of each gene within the genomes of *C. camphora* and *C. kanehirae* were selected to detect and visualize syntenic blocks and collinearity gene pairs using the MCscan pipeline in JCVI [92]. The ratio of *K*_*a*_ to *K*_*s*_ of each gene was estimated using TBtools [93] (v.1.098) with default parameters. Genes with *K*_*a*_*/K*_*s*_ > 1 were considered to evolve under positive selection. Furthermore, SyRI [94] was used to identify structural variations between *C. camphora* and *C. kanehirae* after mapping using Minimap2 [95] (v.2.6).

### Gene predictions and functional annotation

Functional annotation of protein-coding genes was performed using eggNOG-mapper v2 (http://eggnog-mapper.embl.de) with default parameters. The results were described, including Swiss-Prot, GO terms, KEGG pathways, enzyme commission (EC) numbers, and COGs with their associated functional categories. Moreover, BLASTp [96] analyses against protein databases of *Arabidopsis* (Athaliana_167_TAIR10.protein.fa) from Phytozome v13 (https://phytozome-next.jgi.doe.gov) were conducted to assign the best hits (E-value cut-off 1e−5) to these protein-coding genes. TF genes were identified using PlantTFDB 4.0 (http://planttfdb.gao-lab.org).

### RNA-seq and gene expression analysis

The three tissue samples (leaves, stems, and flowers) mentioned above and leaves under different cold acclimation treatments (0, 2, and 12 h) were used to perform RNA-seq on the Illumina X-ten platform. The raw paired-end RNA-seq reads were filtered into clean data by FASTP [97] (v.0.12.4 ). Afterward, HiSAT2 [26] (v.2.2.0) was adopted for the mapping of clean reads to genome assemblies, followed by the analysis of transcripts per kilobase of exon model per million mapped reads (TPM) using StringTie [98] (v.2.1.4) . Gene expression and enrichment were analysed and visualized using TBtools or Cytoscape [99] (v.3.7.1). Pairwise DEGs analysis among the control, CA2, and CA12 was conducted using the R package DESeq2 [100] v.1.12.2with |log_2_FC| >1 and *p*_*adj.*_ < 0.01. Additionally, gene coexpression networks were constructed by weighted correlation network analysis (WGCNA) [101]. Network construction and module detection were conducted using an unsigned TOMtype. Soft threshold power was selected to make the whole network fit the scale-free topology with minModuleSize of 30, and mergeCutHeight of 0.25. Module-trait correlations were calculated by Pearson’s correlation coefficient analysis to select trait-specific modules. Cytoscape (v3.7.1) was applied for network visualization of representative modules.

### Gene family or orthogroup inference

Protein and CDSs of representative plant species were downloaded from open sources: *A.* *trichopoda*, *Ananas comosus*, *Musa acuminata*, *Oryza sativa*, *Sorghum bicolor*, *Citrus clementina*, *A. thaliana*, *Populus trichocarpa*, *Vitis vinifera*, *Coffea canephora*, *Cucumis sativus*, *Phaseolus vulgaris*, and *Solanum lycopersicum* from Phytozome v13 (https://phytozome-next.jgi.doe.gov); *Gnetum montanum*, *Chimonanthus salicifolius*, *C. kanehirae*, *L. chinense*, and *Piper nigrum* from NCBI Genome Database (https://www.ncbi.nlm.nih.gov/genome/); *Ginkgo biloba* from the Ginkgo Database (https://ginkgo.zju.edu.cn/genome/); and *Nymphaea colorata*, *N. thermarum* from the Waterlily Pond (http://waterlily.eplant.org). Gene families or orthologous groups of these species were determined by OrthoFinder [82] (v.2.5.2). The Pfam of each species was calculated from the Pfam website v.34.0 (http://pfam.xfam.org). The Pfam number of each species was transformed into *z* scores, and significant expansions or reductions of Pfams in *C. camphora* were based on a *z* score greater than 1.5 or less than −1.5, respectively. The significant Pfams were sorted by Pfam numbers. Gene family expansion and loss were inferred by gene number ratios between *C. camphora* and other species in each orthogroup.

### Target gene family identification and tandem duplication analysis

Two methods, BLASTp [96] and HMMER search [102], were integrated to identify members of the target gene family. Here, we took the identification of *TPS* genes as an example. Specifically, *C. camphora* homologues in *TPS* gene family were screened by a BLASTp [96] search using protein sequences from the representative *A. thaliana* or Swiss-Prot as queries with an E-value cut-off of 1e−5. Then, Pfam domains of *TPS* gene family (PF01397 and PF03936) were identified in all predicted proteins of *C. camphora* by a HMMER [102] search (v.3.0), and hits with an E-value cut-off of 1e−5 were filtered for the subsequent analysis. The protein sequences retrieved by the two methods were further verified by the NCBI Conserved Domain Database (https://www.ncbi.nlm.nih.gov/cdd) and then manually checked. Afterward, multiple sequence alignment was performed using MAFFT [83] (v.7.467), and maximum likelihood phylogenetic trees were constructed by FastTree [103] (v.2.1.12 ) using the JTT+CAT model. In addition, MCScanX [104] in TBtools was adopted to identify genes generated by tandem duplication.

### Comparisons among *Cinnamomumcamphora* genomes

The MCscan pipeline in JCVI [92] was used for the synteny analyses. Gene families or orthologous groups of 22 samples (three *C. camphora* genomes, one *C. kanehirae* genome, one *C. longepaniculatum* transcriptome, and all 17 available *C. camphora* transcriptomes from different sample collection sites) were determined by OrthoFinder [82]. Data source of these samples is in Additional file 1: Table S3. The species tree was constructed based on single-copy OGs. Then, a pan-genome/transcriptome analysis was performed using the obtained OGs to determine the private and shared gene families of three *C. camphora* genomes with high quality. KEGG enrichment and the *K*_*a*_*/K*_*s*_ values were conducted using the same methods as above.

### Metabolomics analyses

The same tissue samples (leaves, stems, and flowers) collected for RNA-seq were used to detect primary metabolites by UPLC-Q-TOF/MS analysis. The procedures, including metabolite extraction, profiling, and data analysis, were as described by Cai et al. [105]. Furthermore, the identification of metabolites was performed using AMDIS (http://chemdata.nist.gov/) and MS-DIAL [106] software and checked manually. The metabolite content was calculated as peak intensity per sample weight, followed by log transformation and Pareto scaling during the normalization process.

### Detection of volatile compounds by GC‒MS analysis

A headspace solid-phase microextraction (SPME, Supelco, PA, USA) system was employed to collect the volatile compounds from three tissue samples, including leaves, stems, and open flowers, following the method of Wang et al. [56]. Specifically, the volatiles from 1 g of each sample were extracted with SPME fibre at 50 ℃ for 30 min. Total trapped volatiles were subsequently thermally desorbed and transferred to an Agilent 7890B-7000C (Agilent Technologies, CA, USA) GC‒MS equipped with an HP-5MS capillary column (30 m × 0.25 mm). The temperature program was isothermal at 50 ℃ for 2 min, then increased at a rate of 5 ℃ per min to 180 ℃ for 5 min, followed by an increase in the rate by 10 ℃ per min to 250 ℃ for 8 min. Volatile compounds were identified by comparing mass spectra with the NIST 17 mass spectral database.

### Subcellular localization and functional validation of TPSs

For subcellular localization and gene function validation in vivo, coding sequences of *TPS45*, *TPS56*, and *TPS79* were cloned into the pHB vector to obtain constructs of 35S::TPSs-YFP. Then, the empty vector and constructs containing the above *TPSs* were carried by *Agrobacterium tumefaciens* strain GV3101 and infiltrated into tobacco (*Nicotiana benthamiana*) leaves. After 2 days of infiltration, the infiltration point was collected for subcellular localization observation by laser scanning confocal microscope and volatile determination using GC‒MS as described above.

### Variant analysis

Since *C. camphora* is the most dominant tree in the landscape of Hangzhou, China, samples from individuals of different ages ranging from 10 years to over 700 years were randomly selected to detect phylogenetic and genetic variation. In total, DNA from leaf tissues of 74 *C. camphora* accessions and the sample used for genome sequencing were used for the construction of 100-bp paired-end libraries at Beijing Genomics Institute (BGI, Shenzhen, China) and were then sequenced using the BGISEQ-500RS platform. A total of 621.68 Gb of data were generated, yielding an average coverage of 13× per accession. Raw reads were quality controlled using FASTP to remove adaptors and low-quality bases. The clean reads were aligned against the reference genome of *C. camphora* using BWA-MEM with default parameters. Variant detection was performed using the genome analysis toolkit (GATK [107] v.4.1.7.0) following the best practices workflow for variant discovery. Variants were called in each accession separately using HaplotypeCaller, and individual gvcf files were merged using GenotypeGVCFs. This two-step approach includes quality recalibration and regenotyping in the merged vcf file, ensuring variant accuracy. After quality control of the SNPs using VCFtools [108] (v.0.1.16), the SNPs were hard filtered using GATK VariantFiltration (DP<300|| DP>3000|| QD<2.0|| FS>60.0|| MQ<40.0|| MQRankSum < −12.5 || ReadPosRankSum < −8.0). The resulting biallelic SNPs with a minor allele frequency greater than 10% (-m 0.1) and missing rate less than 40% (-M 0.4) were selected for further analysis. The same protocol was applied to the additional analyses of the above 75 accessions using *C. kanehiare* as the outgroup. Only SNPs on Chr1 were taken as representatives.

### Phylogenetic construction, admixture analysis, principal component analysis, and detection of selective sweeps

Before phylogenetic analysis, SNPs in linkage disequilibrium (LD) were dropped out in Plink [109] (v.1.3.0) using a sliding window of 50 SNPs with a 10 SNP step size, and SNPs with correlation coefficients (R^2^) > 0.2 were filtered out. A total of 11.7 million whole-genome SNPs were used for phylogenetic analysis. A NJ phylogenetic tree of all 75 samples was constructed using VCF2Dis (https://github.com/BGI-shenzhen/VCF2Dis) with default parameters. Then, we used MEGAX to show and modify the constructed trees. The admixture analysis was carried out using ADMIXTURE [110] (v.1.3.0), and the optimal number of populations (*K*) was selected using tenfold cross-validation. We used Plink [109] (v.1.3.0 ) for the calculation of principal components and visualized the results using Base R software v.4.0.5 with customized scripts. A genome-wide scan of selective sweeps was performed by comparing allele frequency between groups separated by principal component analysis (PCA). The *F*_*ST*_ and *π* ratios were calculated based on a sliding window of 100 kb and a step size of 10 kb using VCFtools [108] (v.0.1.16) . Regions ranked in the top 5% of the values in both methods were defined as putative selective sweeps. KEGG enrichment of candidate genes in the above regions was carried out using TBtools [93] (v.1.098 ).

### Selection analysis across the whole genome

Genetic diversity (in terms of nucleotide diversity *π*) and Tajima’s *D* at the whole-genome level were observed to assess whether genomic regions were under selection signals. VCFtools [108] (v.0.1.16) was used to calculate genetic statistics across the entire genome with a 100-kp sliding window for *π* and Tajima’s *D* using the SNP data. Windows with Tajima’s *D* value less than an empirical threshold of 5% or greater than the 95% confidence limit were considered to be significant. Windows showing significant positive Tajima’s *D* values were considered under highly balancing selection during the evolution process of *C. camphora* if the *π* values of the windows were very low (less than the empirical threshold of 5%). Candidate genes in the above windows were annotated against the Pfam database to identify the conserved domain and putative gene function.

### Supplementary Information

**Additional file 1:** **Table S1. **Detailed information of tandemly duplicated genes in *Cinnamomum camphora*.            **Table S2.** Summary of repeat contents of *Cinnamomum camphora*.     **Table S3.** Data sources of 29 studied Lauraceae species. **Table S4. **Full name list of the gene abbreviations. **Table S5. **Top 50 biological process enriched GO terms (sorted by corrected p-value BH method) of genes in expanded orthogroups in *Cinnamomum camphora* relative to *C. kanehirae*. **Table S6.** *Terpenoid biosynthesis *(*TPS*) gene family of *Cinnamomum camphora*. **Table S7. **Main aromatic compounds of different tissues in *Cinnamomum camphora*.**Table S8.** List of the 75 resequenced *Cinnamomum camphora* accessions. **Table S9.** Protein-coding genes under strong balancing selection.

**Additional file 2:** **Fig. S1.** Genome-wide analysis of chromatin interactions in the *Cinnamomum camphora* genome using Hi-C data. **Fig. S2.** The genome size estimation of *Cinnamomum camphora* by flow cytometric analysis. **Fig. S3.** Synteny analysis between *Cinnamomum camphora* genome assembly in this study and those in published studies. **Fig. S4.** Syntenic comparison between *Cinnamomum camphora* and *C. kanehirae*. **Fig. S5.** Structural variations between *Cinnamomum camphora* and *C. kanehirae*.**Fig. S6. **The jackknife test of variable importance for *Cinnamomum camphora* and *C. kanehirae*. **Fig. S7.** Pfam analyses between *Cinnamomum camphora* and *C. kanehirae*. **Fig. S8.** Weighted gene co-expression network analysis (WGCNA) of the transcriptomes. **Fig. S9. **Coexpression network of a gene in the circadian rhythm pathway (*RON3*, Ccam01g03083). **Fig. S10.** Phylogenetic tree of *cold shock protein* (*CSP*) gene family. **Fig. S11. **Top 20 significantly expanded orthogroups in *Cinnamomum camphora *sorted by gene count ratio between *C. camphora* and *C. kanehirae*. **Fig. S12. **The analyses of genes under positive selection between *Cinnamomum camphora* and its closely related species. **Fig. S13.** Expression patterns of *CYP450* genes in different organs and cold acclimation treatments. **Fig. S14.** Co-expression network analysis based on a highly expressed *CYP450* gene member (Ccam02g00082). **Fig. S15.** Tandemly duplicated genes in *Cinnamomum camphora* are extremely significantly enriched in phenylpropanoid, flavonoid, and lignin-related pathway. **Fig. S16.** Top 20 enriched GO terms in molecular function of tandem-duplicated genes in *Cinnamomum camphora*. **Fig. S17. **Top 50 metabolites with highest contents in leaf, stem, and flower of *Cinnamomum camphora*. **Fig. S18. **Transcriptome analyses of differentially expressed genes (DEGs) with different cold treatments. **Fig. S19.** Gene expression in the phenylpropanoid metabolic pathway indicates the contribution to cold tolerance in *Cinnamomum camphora*. **Fig. S20. **Phylogenetic analysis and copy numbers in subfamilies of *terpenoid biosynthesis* genes (*TPSs*) during flowering plant evolution. **Fig. S21.** *TPS*-related WGCNA module and GO enrichment of TPS co-expression genes. **Fig. S22. **Volatile compound identification in leaf and stem of *Cinnamomum camphora *by GC-MS analysis. **Fig. S23.** Expression levels of *terpenoid biosynthesis* genes (*TPSs*) in the leaves, stems, and flowers of *Cinnamomum camphora*.**Fig. S24. **Terpenoid biosynthesis gene characterization in *Cinnamomum camphora*. **Fig. S25. **The phylogenetic tree constructed by representative single nucleotide polymorphisms (SNPs) obtained from Chr 1 of 75 resequencing *Cinnamomum* *camphora* accessions using *C. kanehirae* as the outgroup. **Fig. S26. **The species tree based on amino acid sequences of identified single-copy orthogroups (OGs) with a coalescent-based method from 22 samples with *Cinnamomum kanehirae* as the outgroup. **Fig. S27. **Functional comparison analyses of *Cinnamomum camphora *genomes with high quality.

## Data Availability

Raw reads of genome and transcriptome sequencing, and whole-genome resequencing for *C. camphora* have been deposited in the NCBI BioProject database under the accession number PRJNA1000241 (https://www.ncbi.nlm.nih.gov/bioproject/PRJNA1000241) [111]. Genome assembly and gene annotations in this study are available in figshare (https://doi.org/10.6084/m9.figshare.20647452.v1). Additionally, the data source of 29 species for phylogenetic analyses in Fig. 1 are summarized in Additional file 1: Table S3. All data generated or analysed during this study are included in this published article, its supplementary information files and publicly available repositories, and additionally mention the source of all analysed datasets.

## Abbreviations

MEP: 2-C-methyl-D-erythritol 4-phosphate
MVA: Mevalonate
PacBio: Pacific Biosciences
Hi-C: High‐throughput chromosome conformation capture
SNPs: Single-nucleotide polymorphisms
LTR: Long terminal repeat
OGs: Orthologous genes
ML: Maximum likelihood
Chr: Chromosome
ENMs: Ecological niche models
*N*_*e*_: Effective population size
Ma: Million years ago
CSD: Cold shock domain
ANA grade: Amborellales, Nymphaeales, and Austrobaileyales
*K*_*a*_: Nonsynonymous substitution rate
*K*_*s*_: Synonymous substitution rate
UPLC-Q-TOF/MS: Ultra-performance liquid chromatography quadrupole time of flight mass spectrometry
CK: Control
CA2: 2 h cold acclimation under 4 ℃
CA12: 12 h cold acclimation under 4 ℃
DEGs: Differentially expressed genes
PAL: Phenylalanine ammonia-lyase
C4H: Catalyses 4-hydroxylation
4CL: 4-Coumarate-CoA ligase
CHS: Chalcone synthase
CHI: Chalcone isomerase
F3’H: Flavonoid 3’-monooxygenase
F3H: Naringenin 3-dioxygenase
FLS: Flavonol synthase
CSE: Caffeoyl shikimate esterase
HCT: Shikimate O-hydroxycinnamoyltransferase
F5H: Ferulate-5-hydroxylase
GC‒MS: Gas chromatography‒mass spectrometry
NJ: Neighbour-joining
PCA: Principal component analysis
GD: Guangdong Province
HB: Hubei Province
GX: Guangxi Province
JX: Jiangxi Province
FJ: Fujian Province
*F*_*ST*_: Population-differentiation statistics
*π*: Nucleotide diversity
PSMC: Pairwise sequentially Markovian coalescence
PI: Propidium iodide
CV: Coefficient of variation
EC: Enzyme commission
TPM: Transcripts per kilobase of exon model per million mapped reads
WGCNA: Weighted correlation network analysis
SPME: Solid-phase microextraction
LD: Linkage disequilibrium
R^2^: Correlation coefficients
*K*: Optimal number of populations
BGI: Beijing Genomics Institute
*C. camphora*: *Cinnamomum camphora*
*C. kanehirae*: *Cinnamomum kanehirae*
*C. longepaniculatum*: *Cinnamomum longepaniculatum*
*A. trichopoda*: *Amborella trichopoda*
*A. thaliana*: *Arabidopsis thaliana*
*L. chinense*: *Liriodendron chinense*
*N. thermarum*: *Nymphaea thermarum*
