# Supplementary material for: Genome and whole-genome resequencing of Cinnamomum camphora elucidate its dominance in subtropical urban landscapes
Source: BMC Biol. 2023 Sep 12;21:192. doi: 10.1186/s12915-023-01692-1 (PMC10496300; doi:10.1186/s12915-023-01692-1)
Supplement: Supplementary file 2 — Additional file 2: Fig. S1. Genome-wide analysis of chromatin interactions in the Cinnamomum camphora genome using Hi-C data. Fig. S2. The genome size estimation of Cinnamomum camphora by flow cytometric analysis. Fig. S3. Synteny analysis between Cinnamomum camphora genome assembly in this study and those in published studies. Fig. S4. Syntenic comparison between Cinnamomum camphora and C. kanehirae. Fig. S5. Structural variations between Cinnamomum camphora and C. kanehirae.Fig. S6. The jackknife test of variable importance for Cinnamomum camphora and C. kanehirae. Fig. S7. Pfam analyses between Cinnamomum camphora and C. kanehirae. Fig. S8. Weighted gene co-expression network analysis (WGCNA) of the transcriptomes. Fig. S9. Coexpression network of a gene in the circadian rhythm pathway (RON3, Ccam01g03083). Fig. S10. Phylogenetic tree of cold shock protein (CSP) gene family. Fig. S11. Top 20 significantly expanded orthogroups in Cinnamomum camphora sorted by gene count ratio between C. camphora and C. kanehirae. Fig. S12. The analyses of genes under positive selection between Cinnamomum camphora and its closely related species. Fig. S13. Expression patterns of CYP450 genes in different organs and cold acclimation treatments. Fig. S14. Co-expression network analysis based on a highly expressed CYP450 gene member (Ccam02g00082). Fig. S15. Tandemly duplicated genes in Cinnamomum camphora are extremely significantly enriched in phenylpropanoid, flavonoid, and lignin-related pathway. Fig. S16. Top 20 enriched GO terms in molecular function of tandem-duplicated genes in Cinnamomum camphora. Fig. S17. Top 50 metabolites with highest contents in leaf, stem, and flower of Cinnamomum camphora. Fig. S18. Transcriptome analyses of differentially expressed genes (DEGs) with different cold treatments. Fig. S19. Gene expression in the phenylpropanoid metabolic pathway indicates the contribution to cold tolerance in Cinnamomum camphora. Fig. S20. Phylogenetic analysis and copy n [file 12915_2023_1692_MOESM2_ESM.pdf]

Genome and whole-genome resequencing of *Cinnamomum camphora* elucidate its dominance in subtropical urban landscapes

Li et al.

Supplementary Information

Additional file 2.

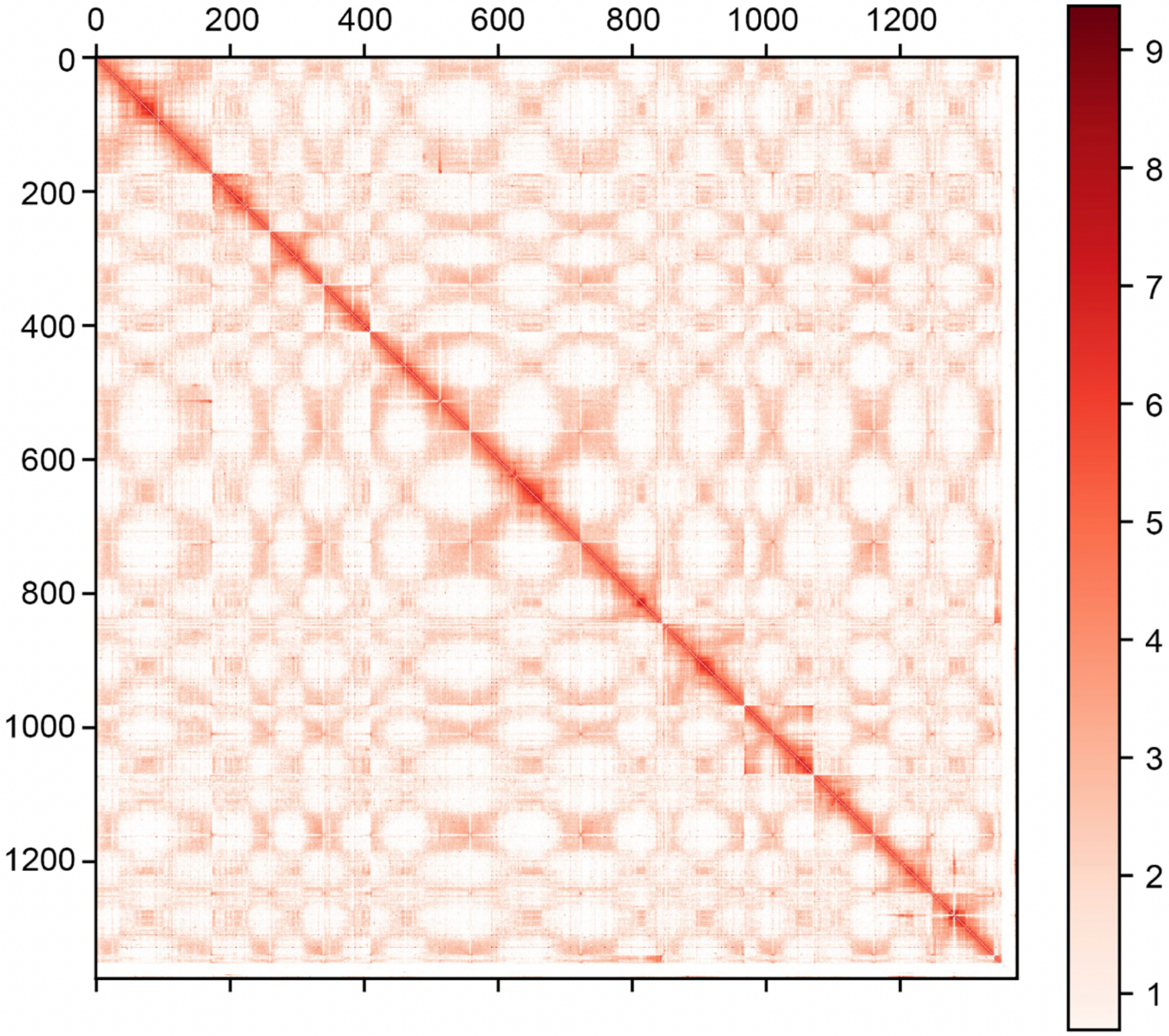

Fig. S1. Genome-wide analysis of chromatin interactions in the *Cinnamomum camphora* genome using Hi-C data. Hi-C reads were realigned back to the assembly and the mappings were converted to the dot intensity indicating the loci collocate in the nucleus. The resolution is 500 Kb.

a

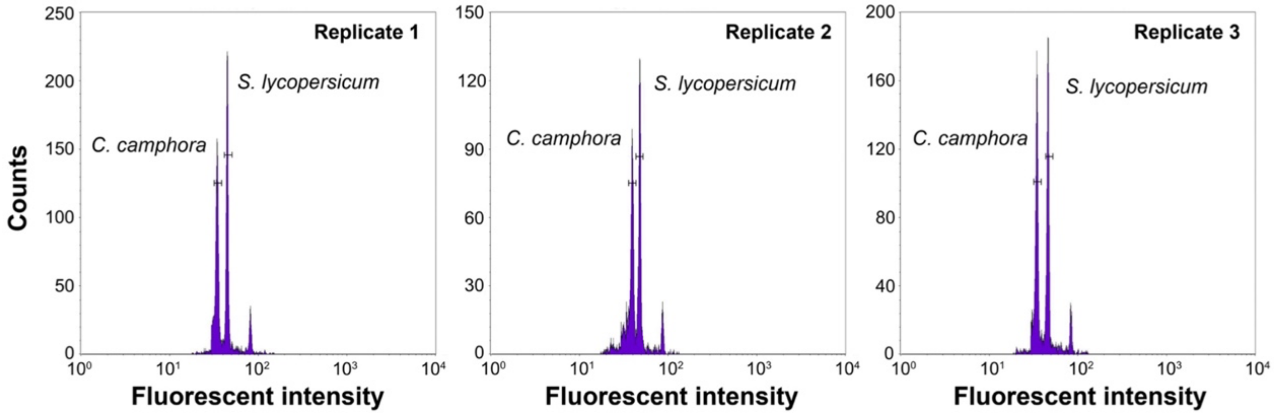

b

Sample ID	Internal standard (IS)	Fluorescent intensity of IS	Fluorescent intensity of sample	Ratio	Estimated genome size (Mb)
1	*S. lycopersicum*	44.05	33.82	0.77	690.99
2	*S. lycopersicum*	47.02	36.47	0.78	698.06
3	*S. lycopersicum*	45.43	33.93	0.75	672.18

Fig. S2. The genome size estimation of *Cinnamomum camphora* by flow cytometric analysis. (a) Flow cytometry estimation of *C. camphora* with three replicates using tomato (*Solanum lycopersicum*) as the internal standard (900 Mb). (b) Fluorescent intensity obtained in the experiment (n = 3) and the estimated genome size of *C. camphora* (687.08 ± 13.38 Mb).

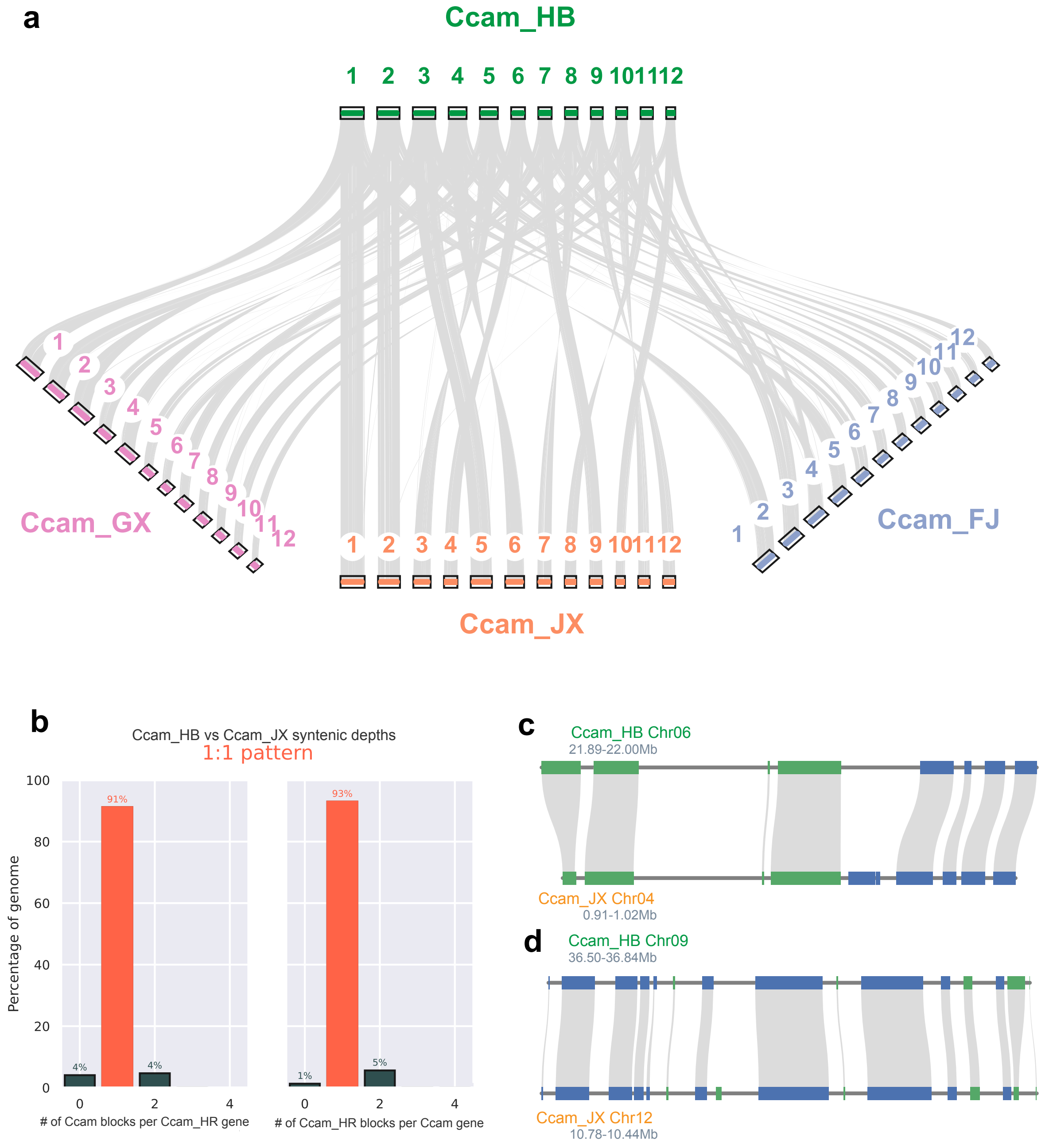

Fig. S3. Synteny analysis between *Cinnamomum camphora* genome assembly in this study and those in published studies. To facilitate the comparison, the genomes of our study and three published ones (Sun et al. 2022 [22]; Jiang et al. 2022 [23]; Wang et al. 2022 [24]) were assigned as Ccam_HB, Ccam_FJ, Ccam_GX, and Ccam_JX, respectively. (a) Syntenic comparison between *C. camphora* genome assembly in this study (Ccam_HB) with three published *C. camphora* genomes. (b) The syntenic depth between Ccam_HB and Ccam_JX. (c, d) Two synteny regions between Ccam_HB and Ccam_JX were randomly selected for visualization.

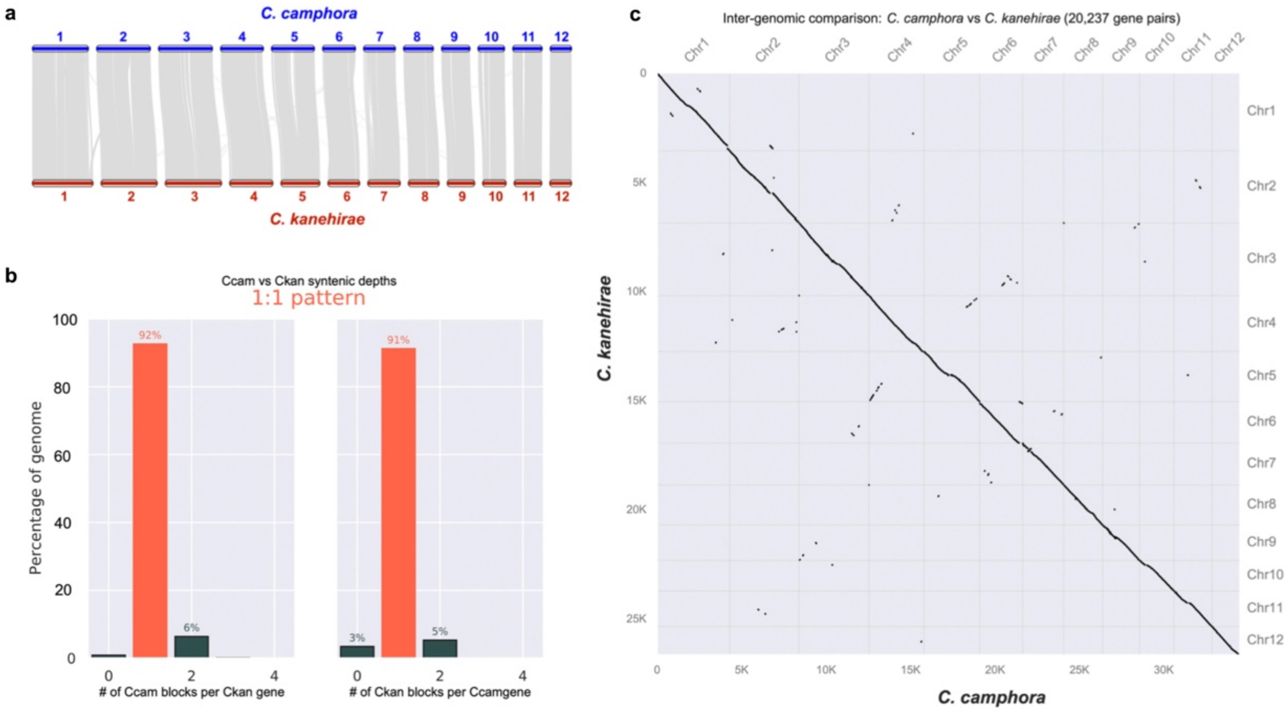

Fig. S4. Syntenic comparison between *Cinnamomum camphora* and *C. kanehirae.* (a) Syntenic comparison between *C. camphora* and *C. kanehirae*. (b) The syntenic depth between two species. (c) Whole-genomic dot plot showing the syntenic relationships between two species.

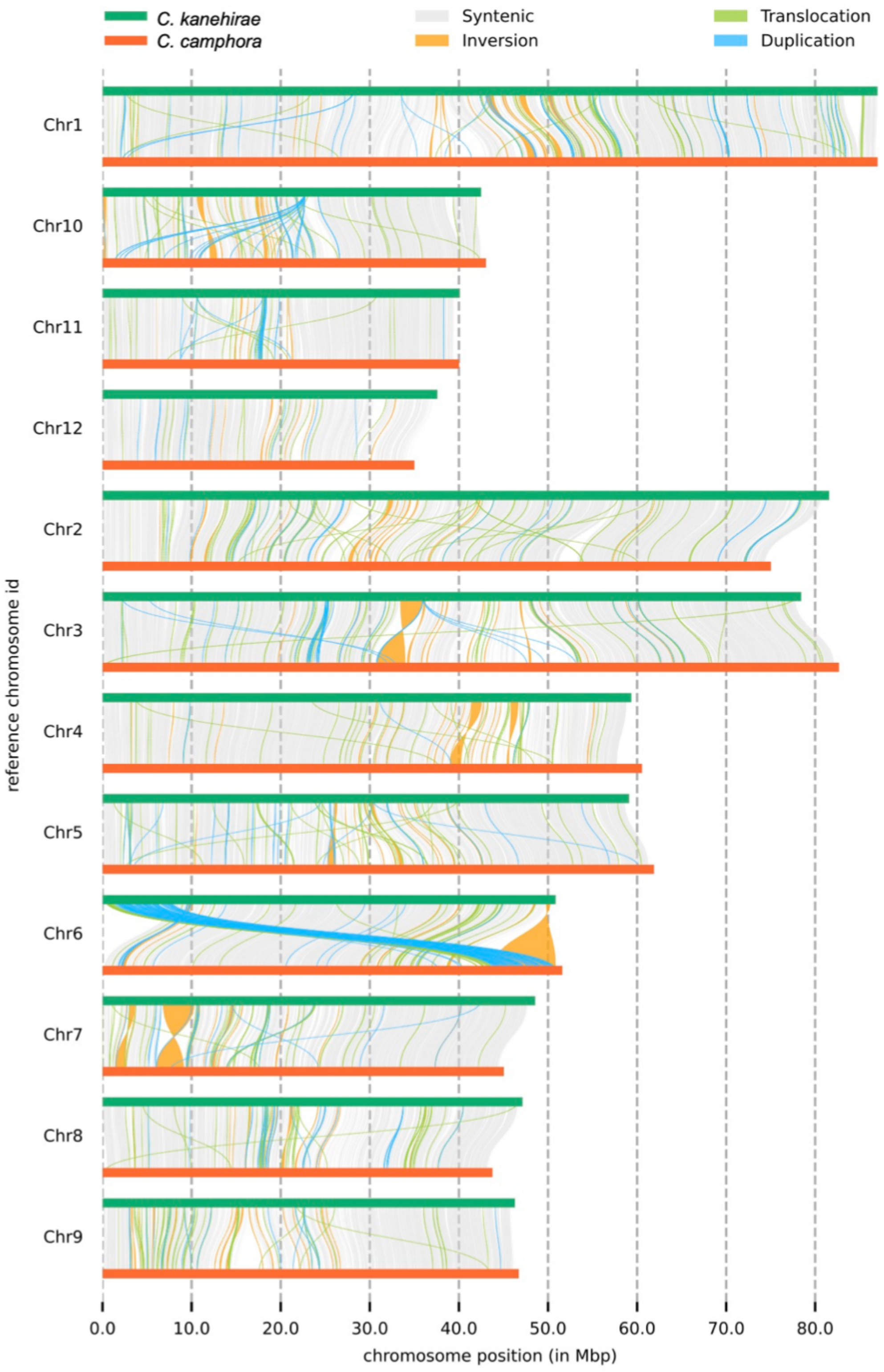

Fig. S5. Structural variations between *Cinnamomum camphora* and *C. kanehirae.*

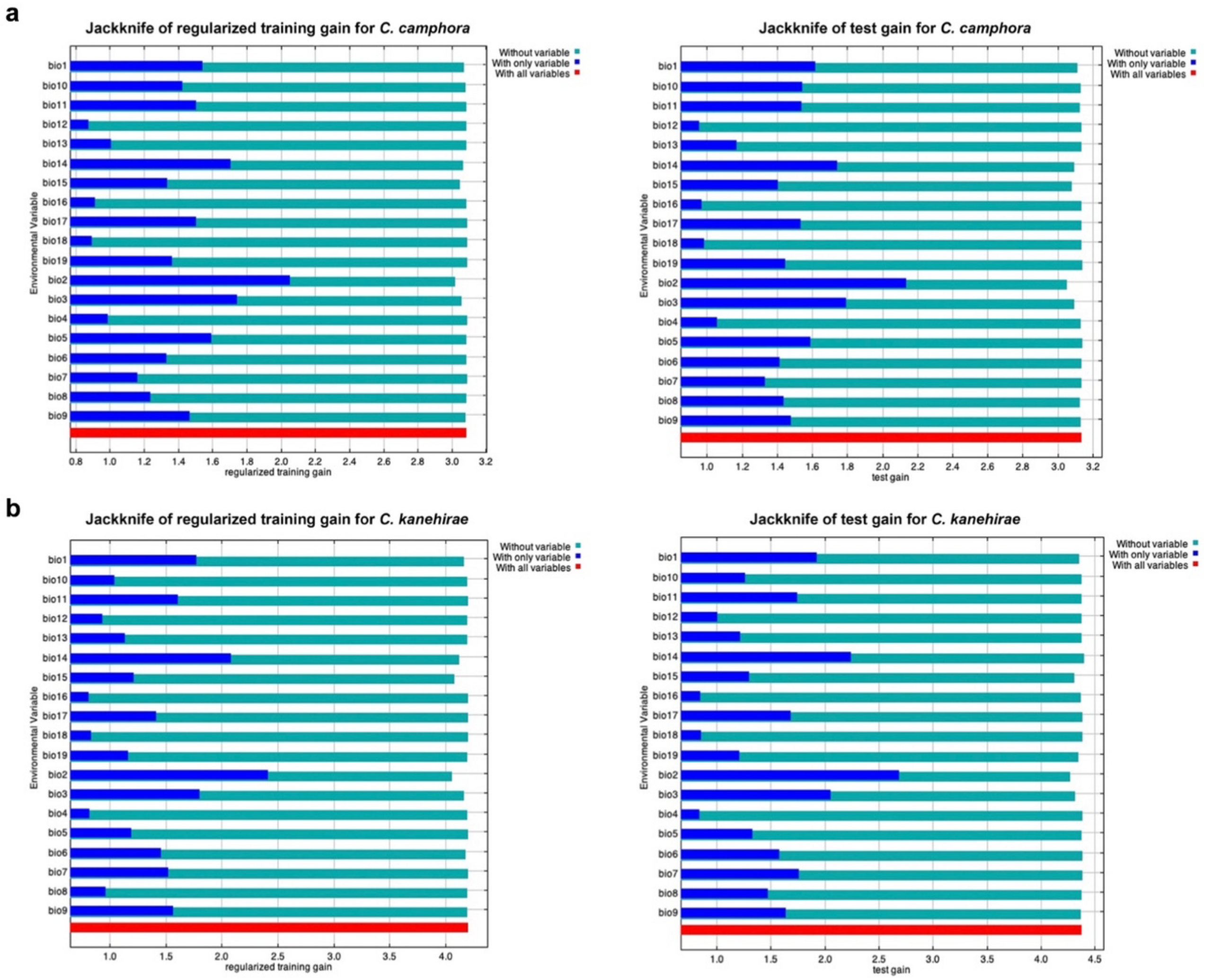

Fig. S6. The jackknife test of variable importance for *Cinnamomum camphora* and *C. kanehirae*. (a) The results of the jackknife test of variable importance for *C. camphora*. For both the training and test data, the environmental variable with the highest gain when used in isolation is bio2 (mean diurnal range (mean of monthly (max. temperature - min. temperature))). (b) The results of the jackknife test of variable importance for *C. kanehirae*. For both the training and test data, the environmental variable with the highest gain when used in isolation is bio2 (mean diurnal range (mean of monthly (max. temperature - min. temperature))), which therefore appears to have the most useful information by itself. The environmental variable that decreases the gain the most when it is omitted is bio2, which therefore appears to have the most information that isn't present in the other variables. Values shown are averages over replicate runs.

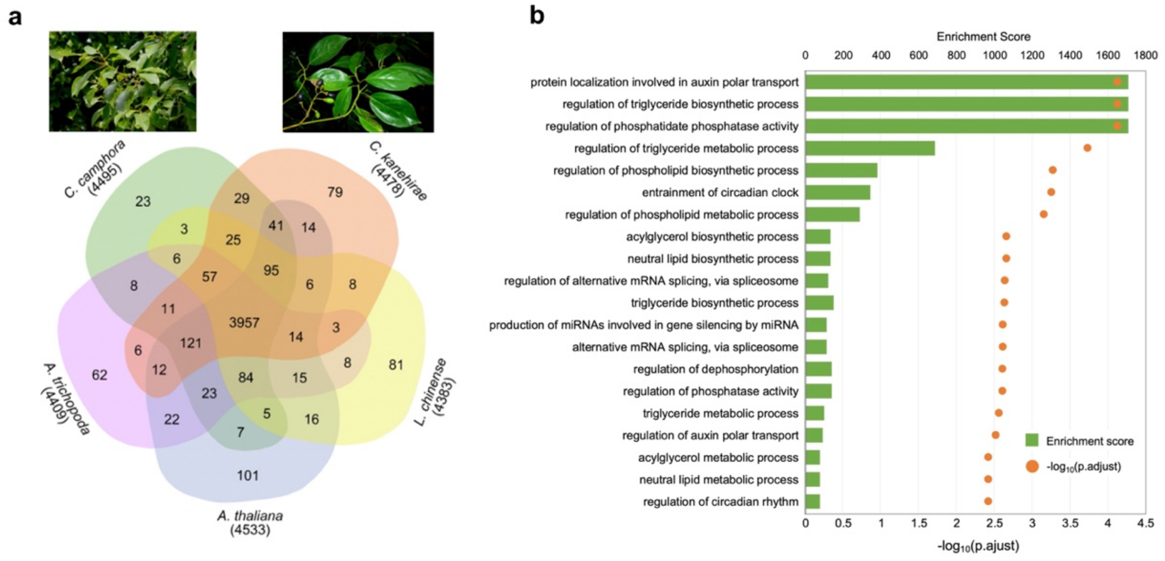

Fig. S7. Pfam analyses between *Cinnamomum camphora* and C. *kanehirae*. (a) Unique and shared Pfams between *C. camphora*, *C. kanehirae*, *Liriodendron chinense*, *Amebralla trichopoda*, and *Arabidopsis thaliana*. The number of Pfams is listed in each of the diagram components, and the total number for each plant is provided in parentheses. The top left panel of the Venn diagram is an image of *C. camphora,* and the top right panel is *C. kanehirae*. (b) Top 20 enriched GO terms of genes in unique Pfams of *C. camphora.*

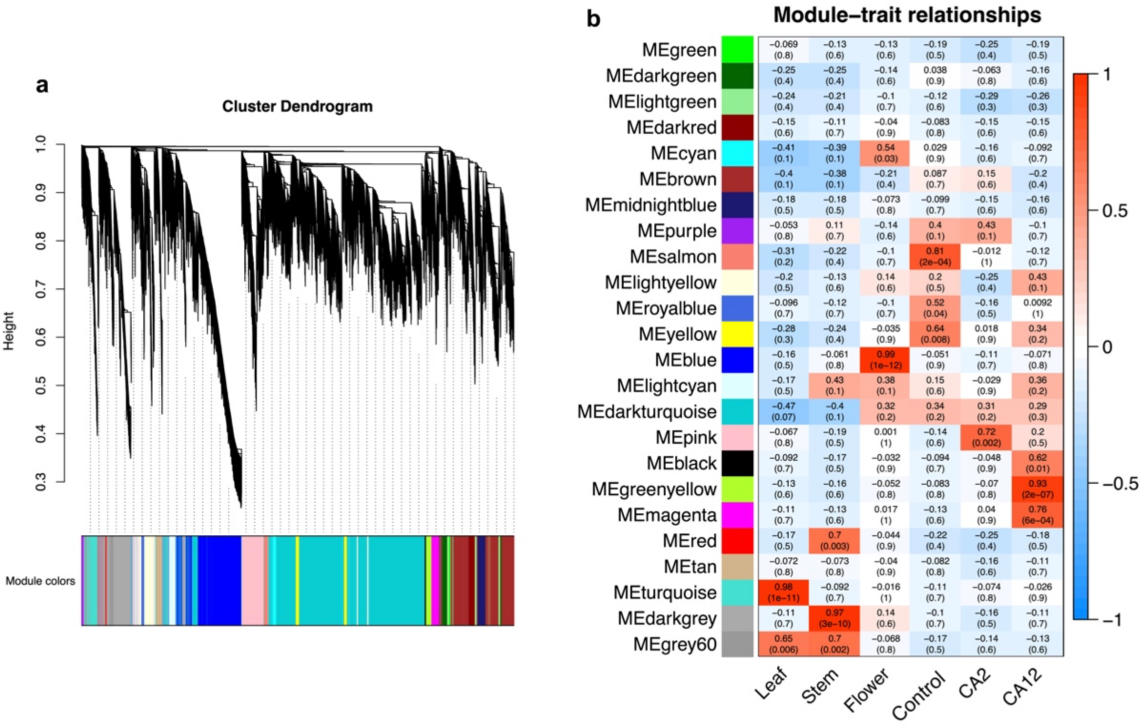

Fig. S8. Weighted gene co-expression network analysis (WGCNA) of the transcriptomes. (a) Clustering dendrogram of genes and merged module colors. In the dendrogram, each leaf corresponds to a gene. (b) Trait-module relationships in the *C. camphora* transcriptomes. Each row represents a module eigengene; each column represents seed composition trait. Each cell contains the corresponding correlation and *p* value. The table is colour coded by correlation according to the color legend.

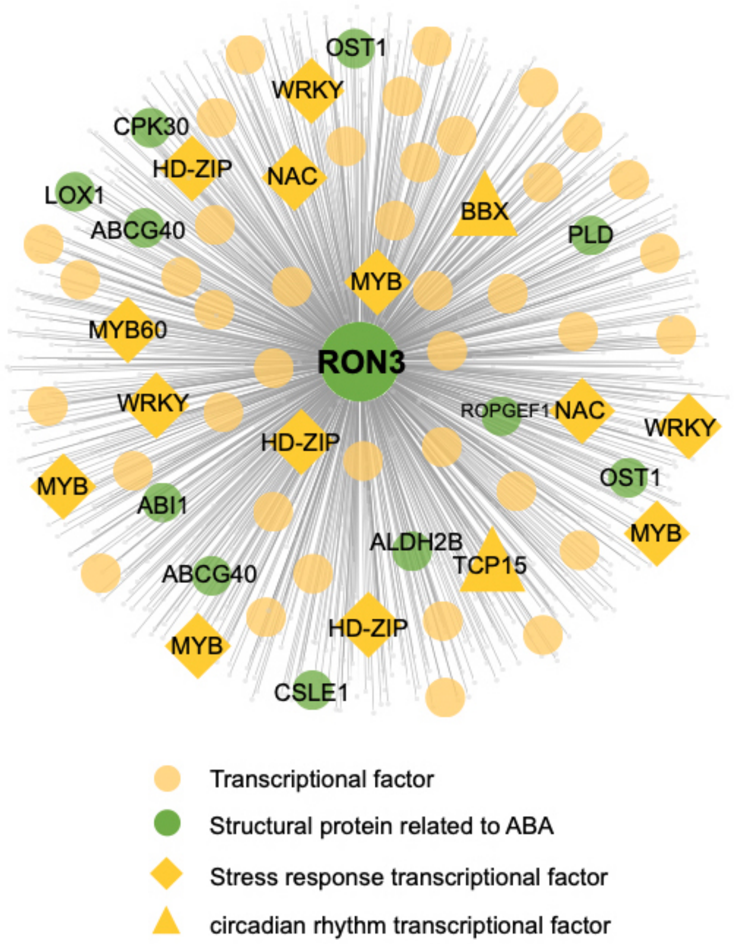

Fig. S9. Coexpression network of a gene in the circadian rhythm pathway (*RON3*, Ccam01g03083). This gene is in unique Pfams of *C. camphora* and enriched in several GO terms.

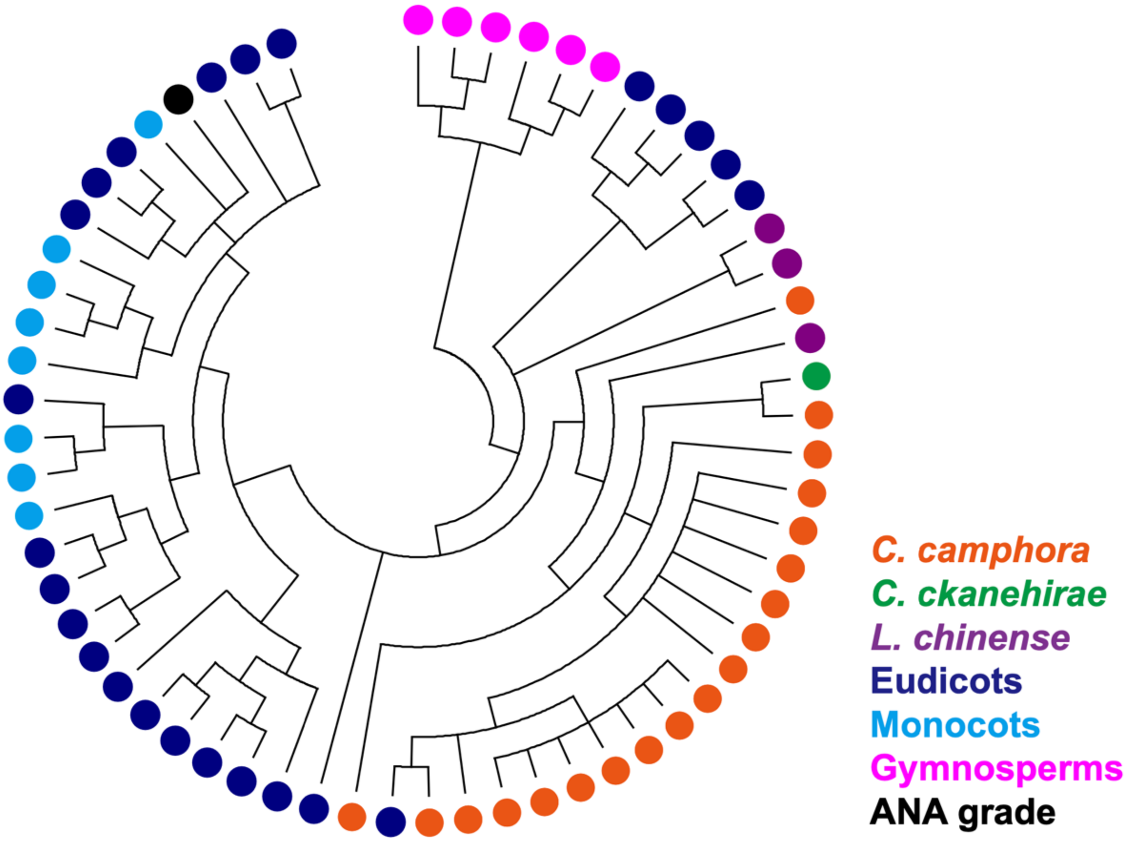

Fig. S10. Phylogenetic tree of *cold shock protein* (*CSP)* gene family. Colour-coded dots in each branch represent different species. Gymnosperms include *Ginkgo biloba* and *Gnetum montanum*. ANA grade includes *Amborella trichopoda*. Eudicots include *Arabidopsis thaliana*, *Citrus clementina*, *Cucumis sativus*, *Populus trichocarpa*, *Solanum lycopersicum*, and *Vitis vinifera*. Monoctos include *Ananas comosus*, *Oryza sativa*, and *Sorghum bicolor*.

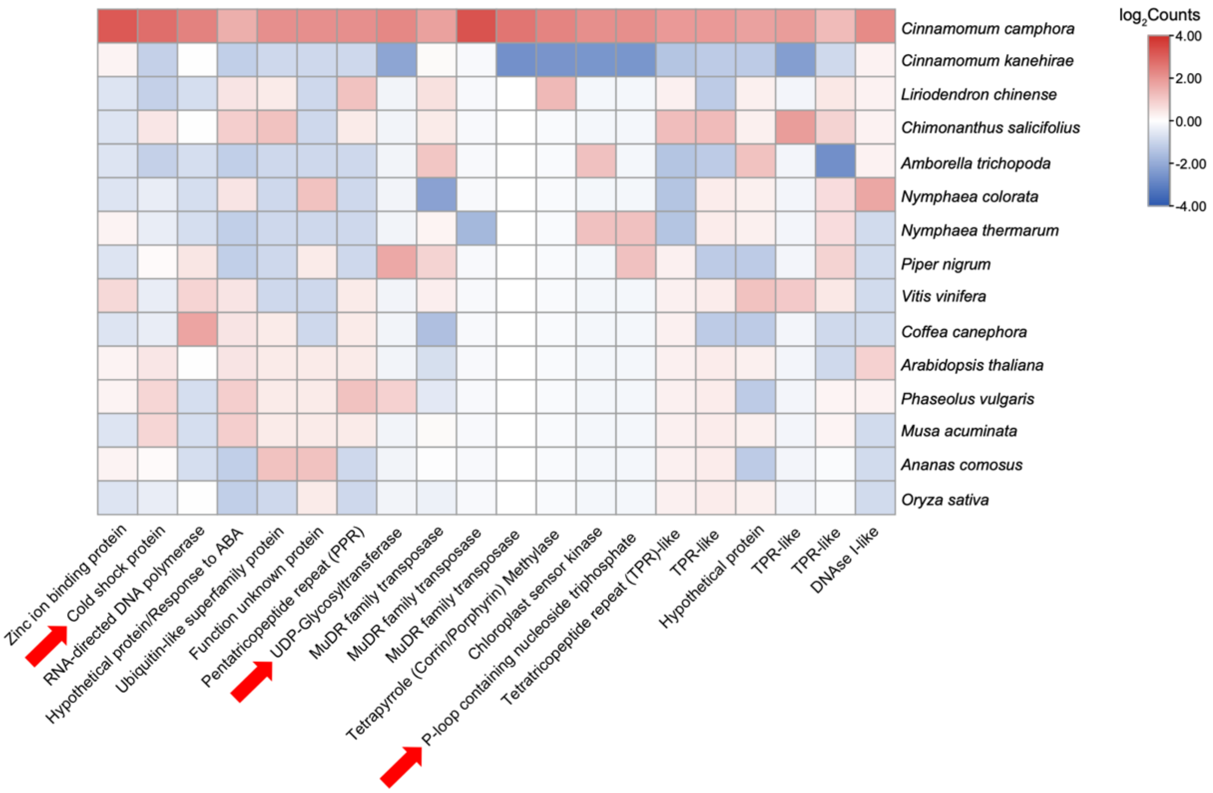

Fig. S11. Top 20 significantly expanded orthogroups in *Cinnamomum camphora* sorted by gene count ratio between *C. camphora* and *C. kanehirae*. For every orthogroup, a z-score was calculated for the corresponding abundance in each species. Only a z-score greater than 2.0 was considered significantly expanded in *C. camphora*. *Arabidopsis* gene function/family was presented on X-axis for each orthogroup.

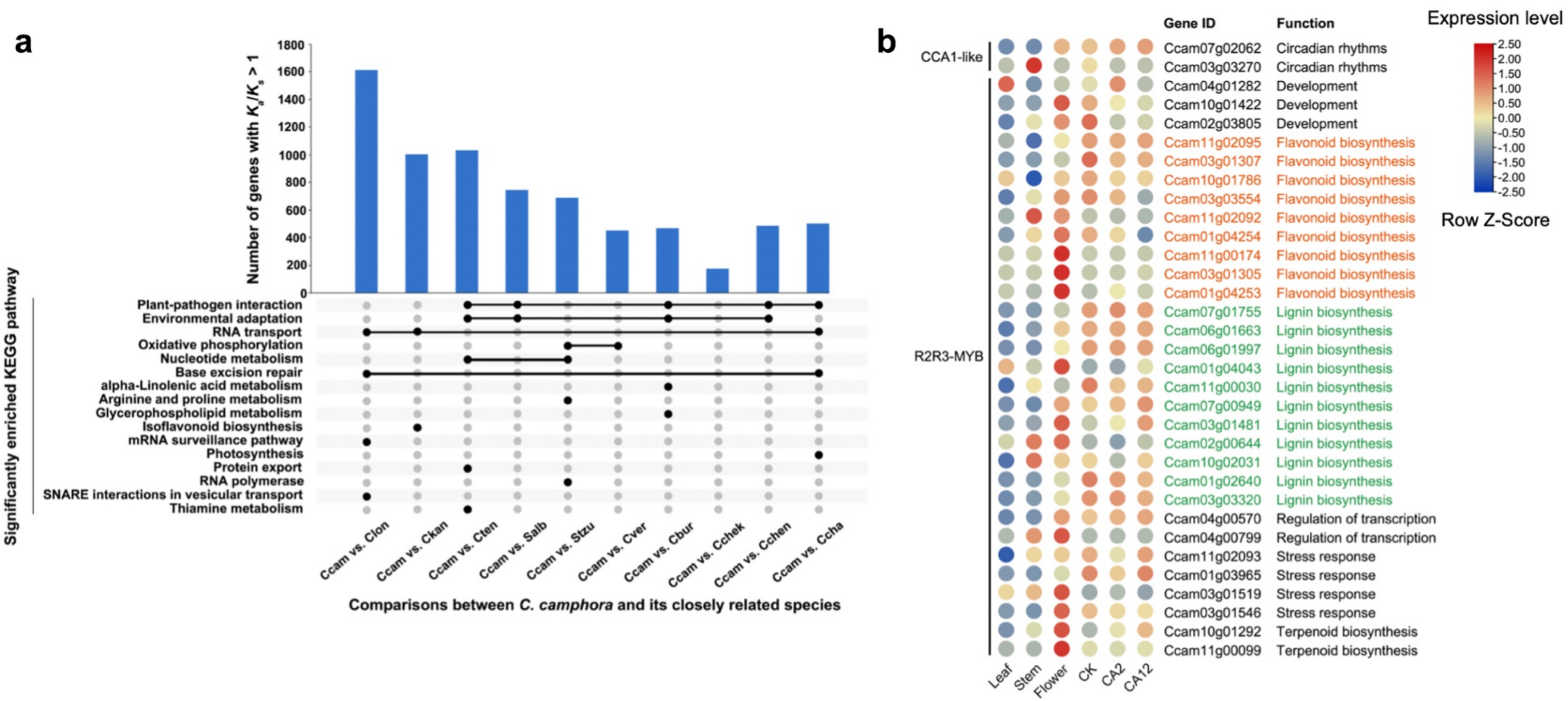

Fig. S12. The analyses of genes under positive selection between *Cinnamomum camphora* and its closely related species. (a) The gene number and significantly (p value < 0.05) enriched KEGG pathways of genes under positive selection ($K_a/K_s > 1$) between *C. camphora* and the 10 most closely related species to *C. camphora* based on the phylogenetic tree in Fig.1a. Ccam, *C. camphora*; Clon, *C. longepaniculatum*; Ckan, *C. kanehirae*; Cten, *C. tenuipile*; Salb, *S. albidum*; Stzu, *S. tzumu*; Cver, *C. verum*; Cbur, *C. burmanni*; Cchek, *C. chekiangense*; Cchen, *C. chennii*; Ccha, *C. chago*. The black dots indicate gene enrichment in corresponding KEGG pathways. (b) Expression patterns and corresponding function of genes in the most enriched gene family MYB. Only the sum of gene expression in three tissues greater than 1.0 is presented in this heatmap.

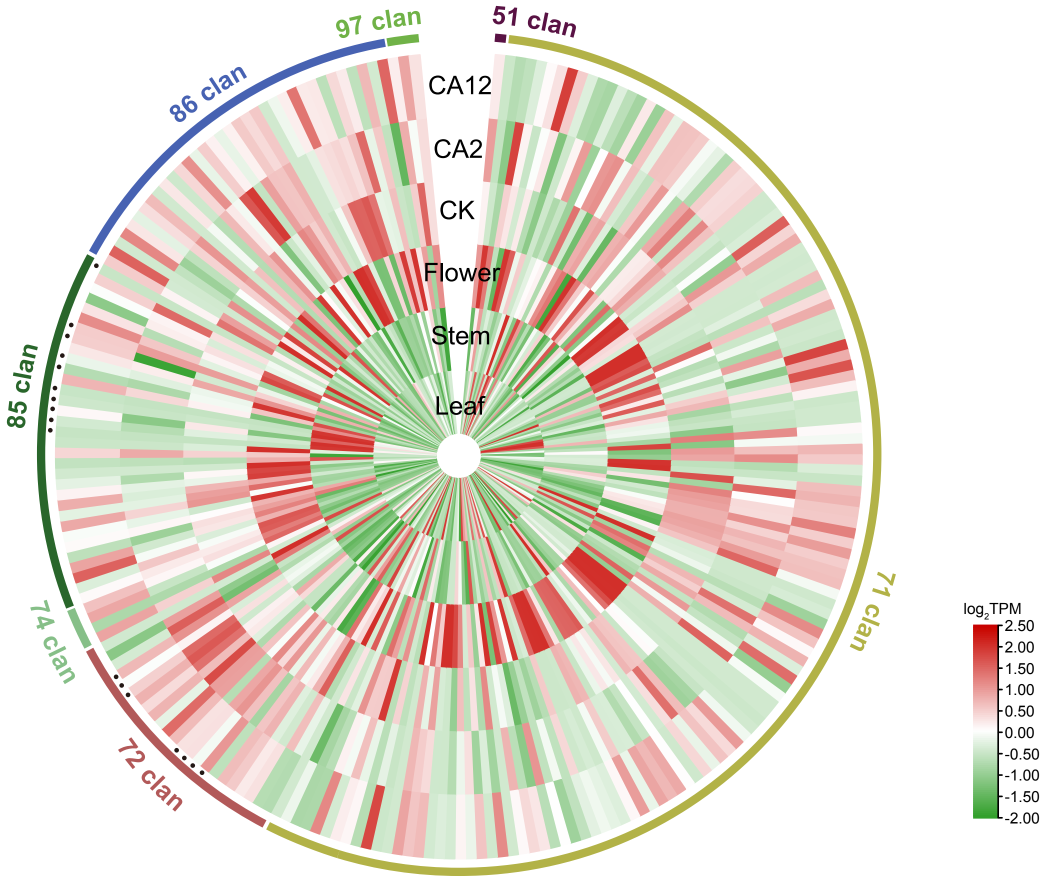

Fig. S13. Expression patterns of *CYP450* genes in different organs and cold acclimation treatments. Control (CK), plants without any treatment; CA2, 2-hour treatment under 4 °C cold acclimation; CA12, 12-hour treatment under 4 °C cold acclimation. Only the sum of gene expression in three tissues greater than 1.0 is presented in this heatmap. Genes marked with black dots were tandem repeats visualized in Fig. 2d.

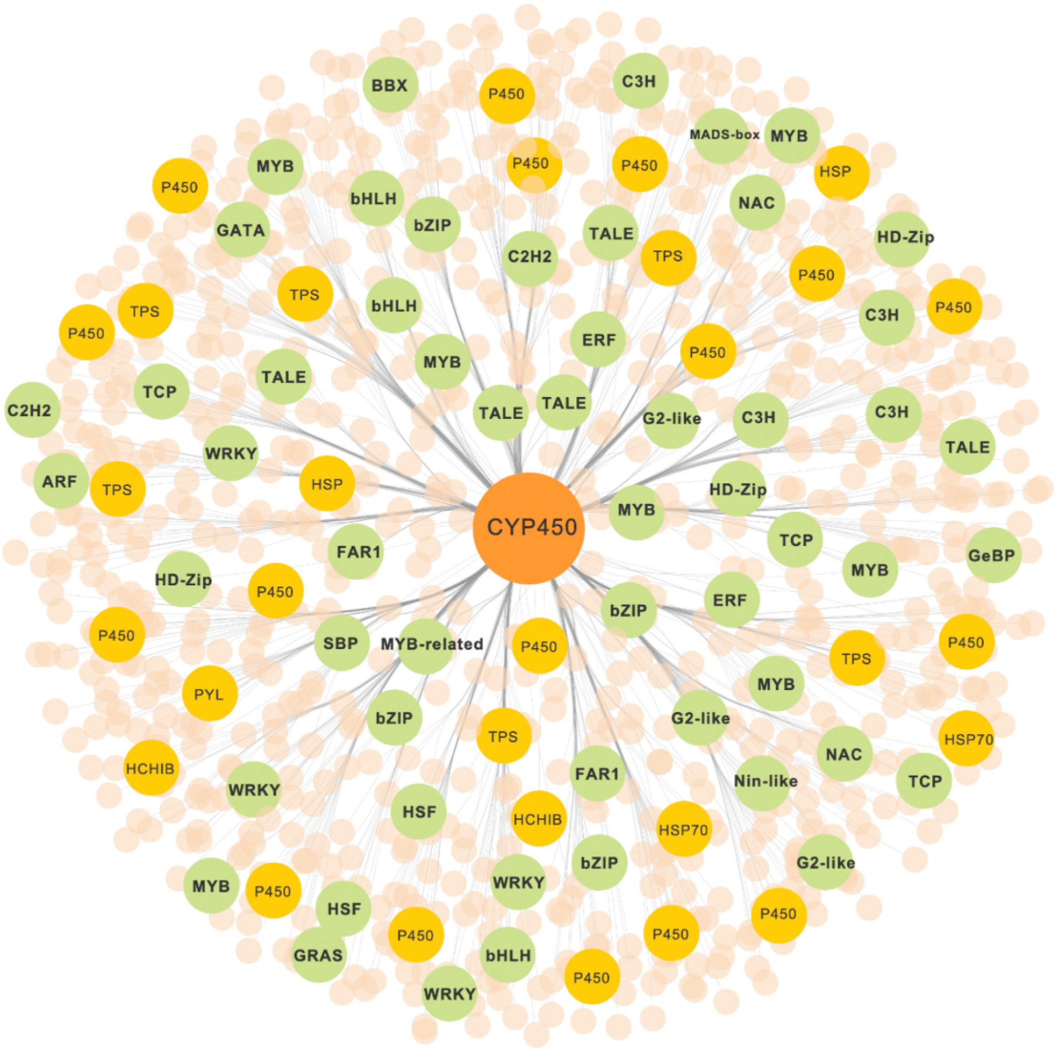

Fig. S14. Co-expression network analysis based on a highly expressed *CYP450* gene member (Ccam02g00082). Orange circles represent functional proteins and green circles represent transcriptional factors.

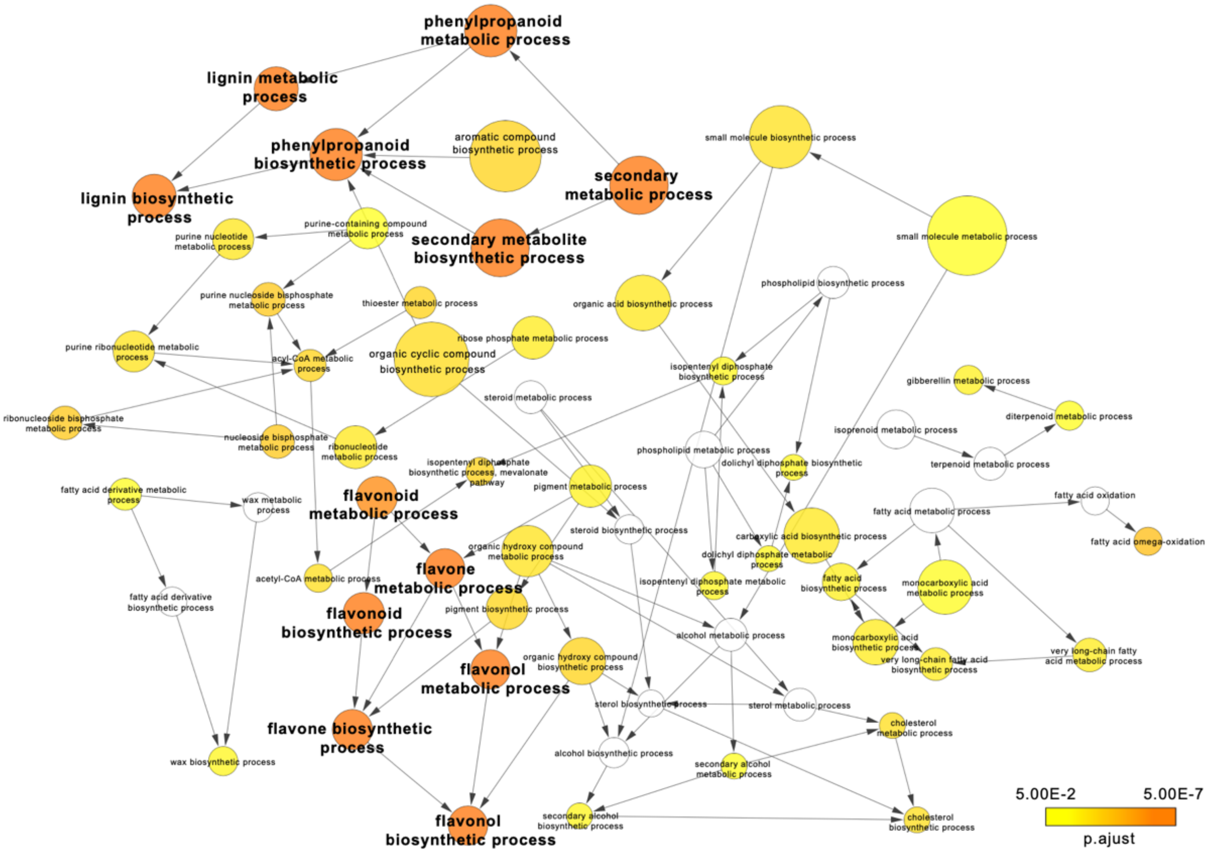

Fig. S15. Tandemly duplicated genes in *Cinnamomum camphora* are extremely significantly enriched in phenylpropanoid, flavonoid, and lignin-related pathway. Circles represent enriched GO terms and are color-coded according to the adjusted p-value. The line with an arrow means the hierarchical level between GO terms.

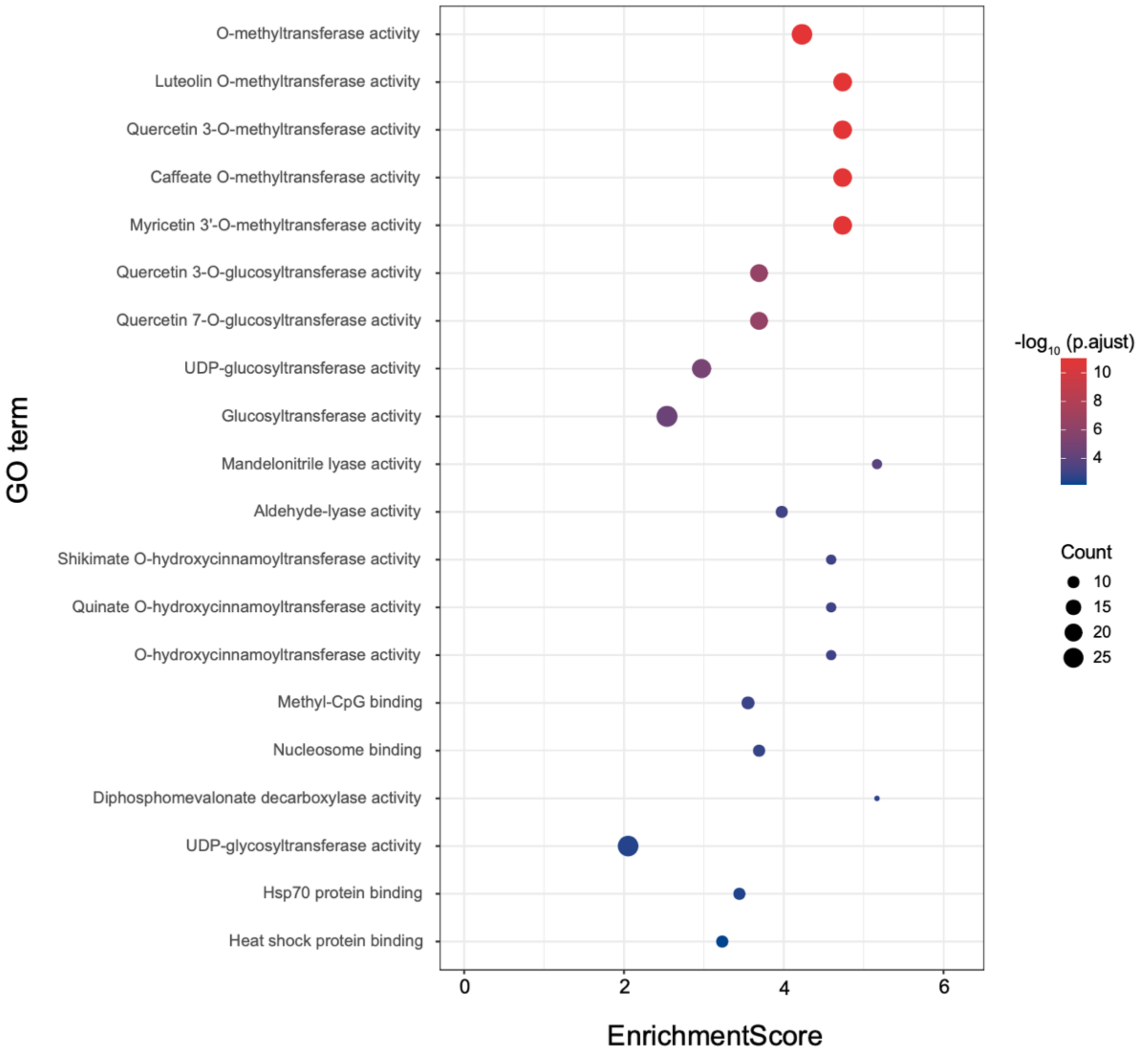

Fig. S16. Top 20 enriched GO terms in molecular function of tandem-duplicated genes in *Cinnamomum camphora*.

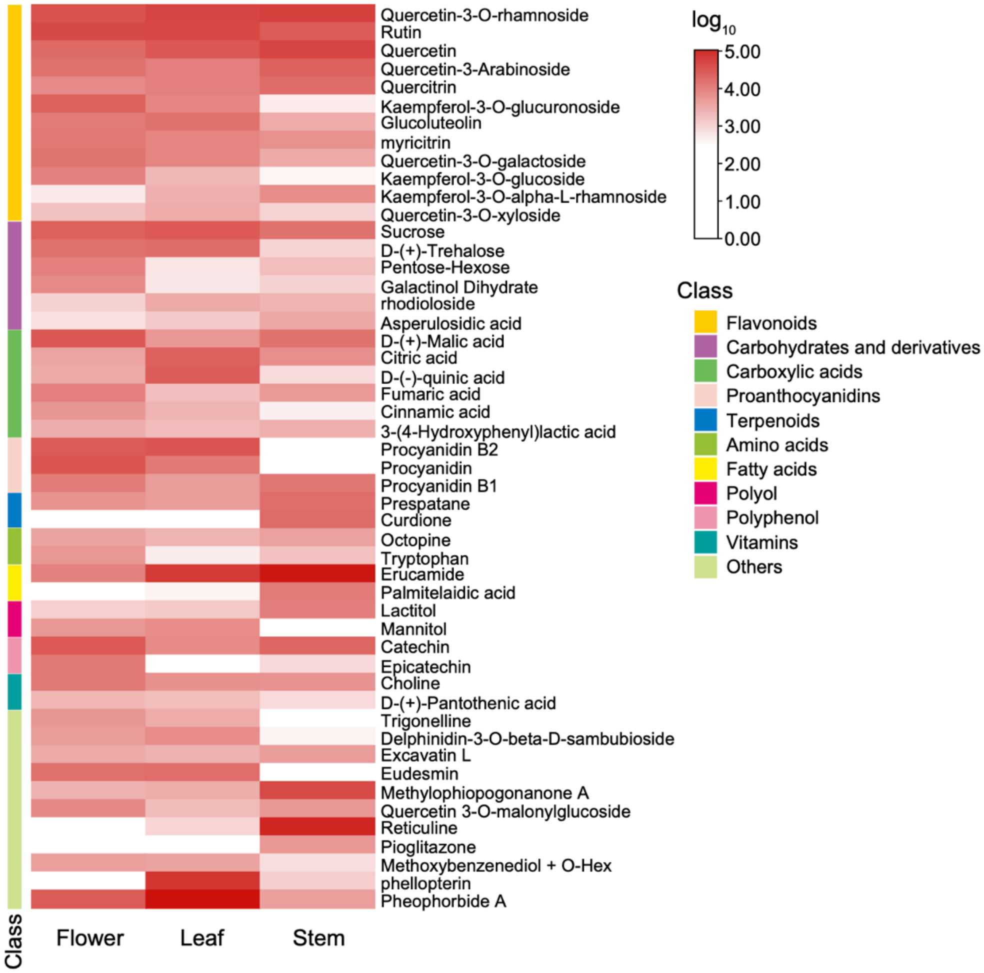

Fig. S17. Top 50 metabolites with highest contents in leaf, stem, and flower of *Cinnamomum camphora*. The most abundant metabolites are flavonoids.

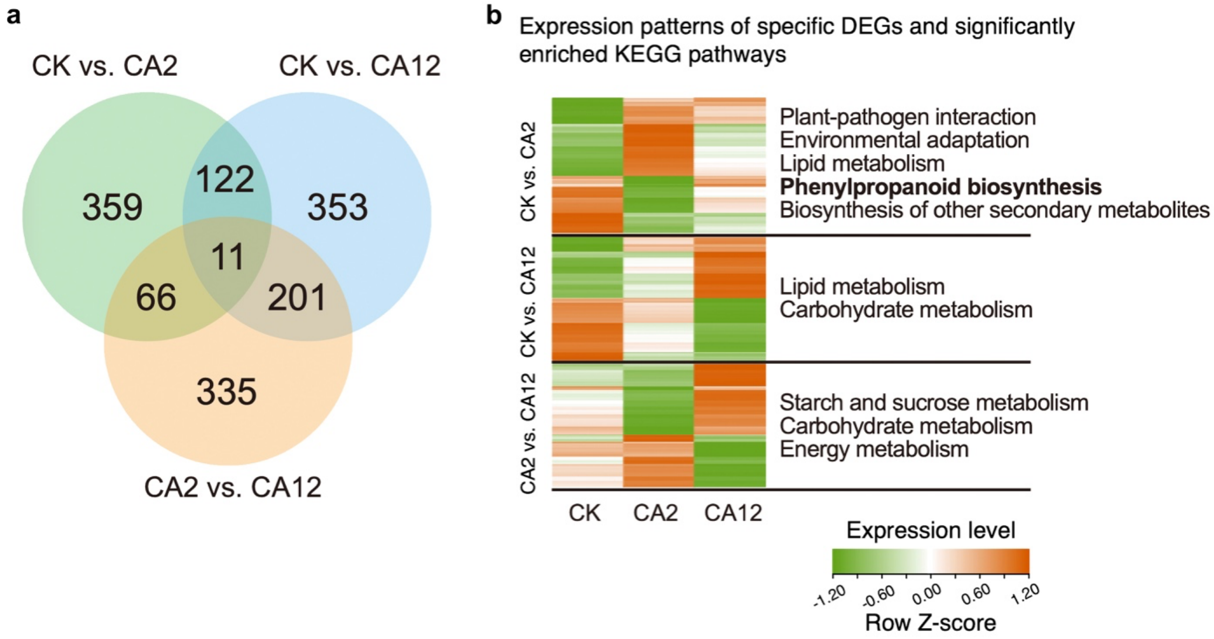

Fig. S18. Transcriptome analyses of differentially expressed genes (DEGs) with different cold treatments. (a) Venn diagram of DEGs obtained from any two treatments. Control (CK), plants without any treatment; CA2, 2-hour treatment under 4 °C cold acclimation; CA12, 12-hour treatment under 4 °C cold acclimation. (b) Gene expression patterns and significantly ($p_{adj.}$ value) enriched KEGG pathways of specific DEGs obtained from any two treatments.

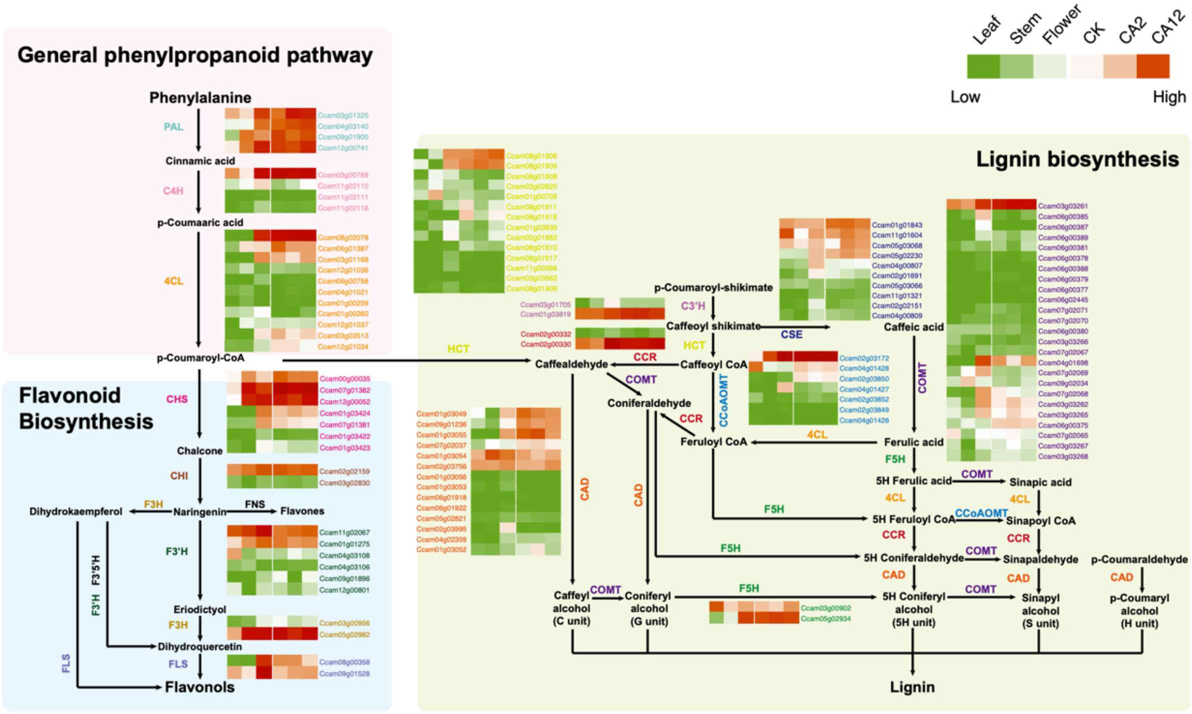

Fig. S19. Gene expression in the phenylpropanoid metabolic pathway indicates the contribution to cold tolerance in *Cinnamomum camphora.* The name of an enzyme is in the same colour as that of its encoding genes.

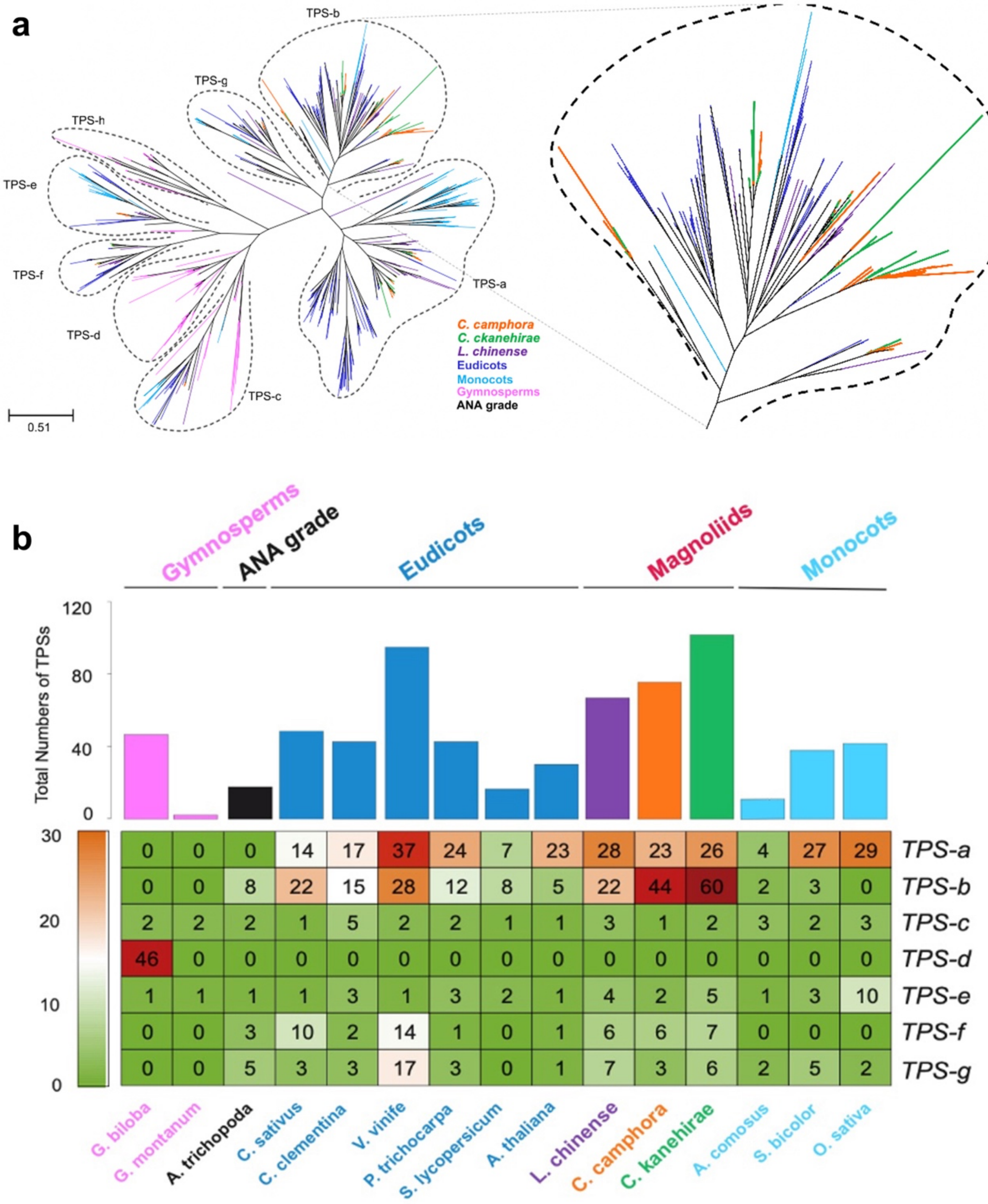

Fig. S20. Phylogenetic analysis and copy numbers in subfamilies of *terpenoid biosynthesis* genes (*TPSs*) during flowering plant evolution. (a) Phylogenetic analysis of *TPSs*. (b) Variation in copy numbers of TPS subfamilies. Gymnosperms include *Ginkgo biloba* and *Gnetum montanum*. ANA grade includes *Amborella trichopoda*. Eudicots include *Arabidopsis thaliana*, *Citrus clementina*, *Cucumis sativus*, *Populus trichocarpa*, *Solanum lycopersicum*, and *Vitis vinifera*. Magnoliids include *Liriodendron chinense*, *C. camphora*, and *C. kanehirae*. Monoctos include *Ananas comosus*, *Oryza sativa*, and *Sorghum bicolor*.

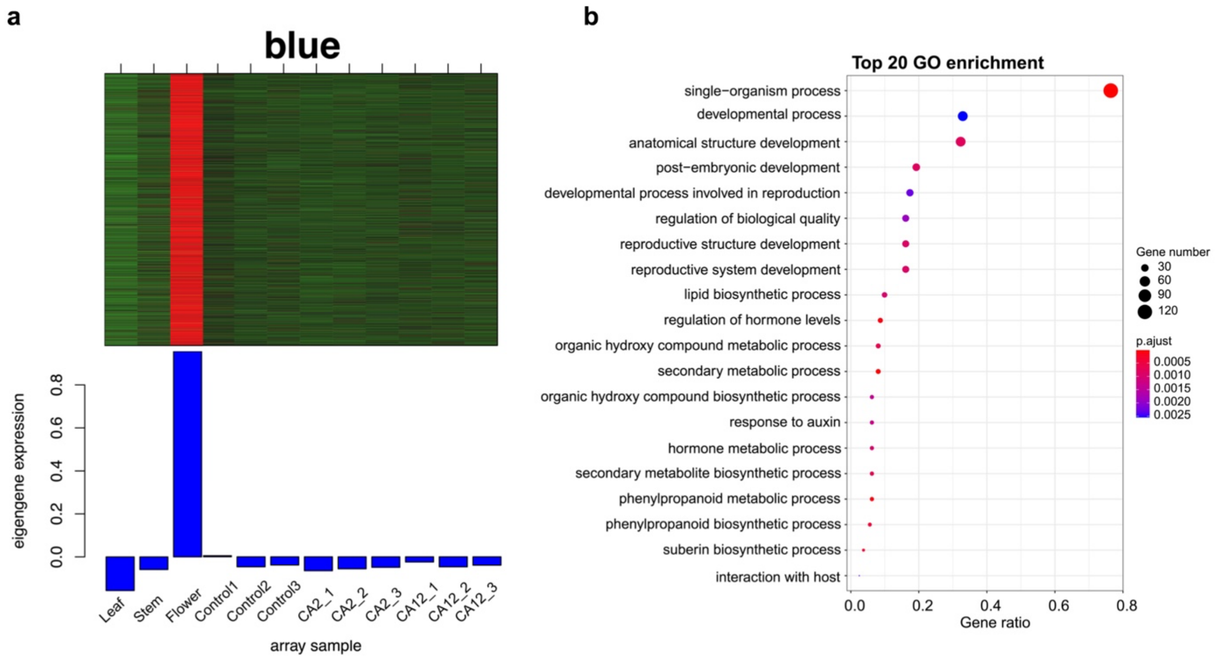

Fig. S21. *TPS*-related WGCNA module and GO enrichment of *TPS* co-expression genes. (a) The heatmap of all genes and the eigengene expression pattern in the blue module, which was significantly correlated with flowers. (b) Top 20 enriched GO terms in biological process of co-expressed genes with *TPSs*.

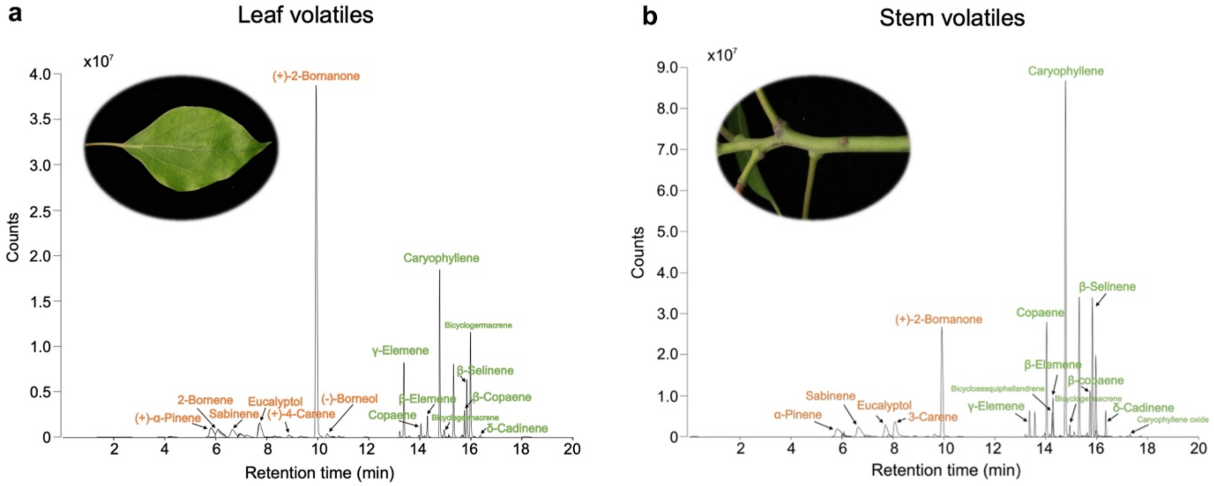

Fig. S22. Volatile compound identification in leaf and stem of *Cinnamomum camphora* by GC-MS analysis. (a) Volatile compounds in leaf. (b) Volatile compounds in stem. Compounds shown in orange are monoterpenes, green are sesquiterpenes, and those in black are non-terpene compounds.

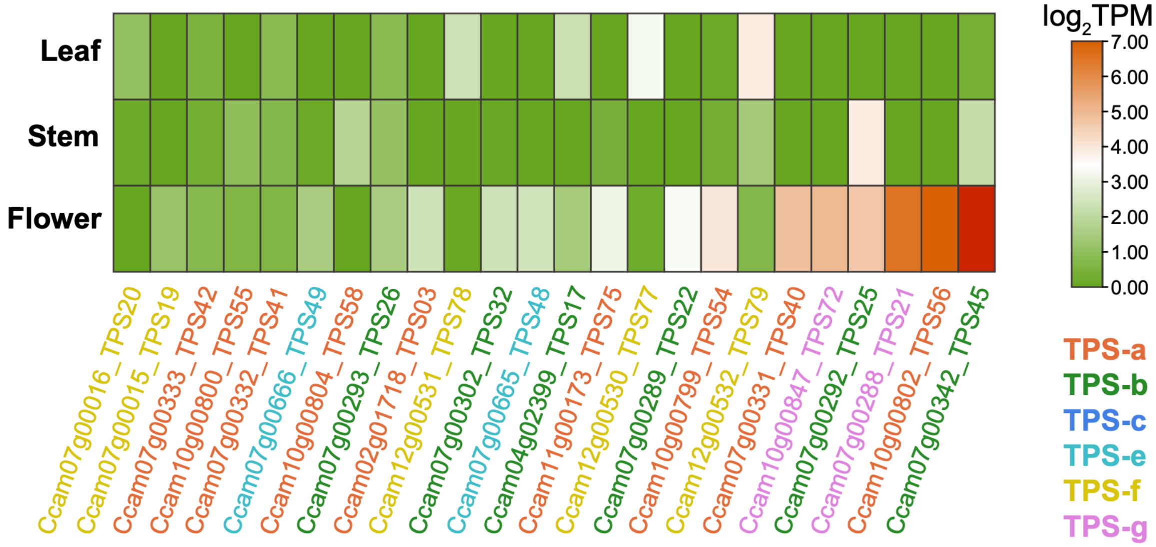

Fig. S23. Expression levels of *terpenoid biosynthesis* genes (*TPSs*) in the leaves, stems, and flowers of *Cinnamomum camphora*. Only the sum of gene expression in three tissues greater than 1.0 is presented in this heatmap.

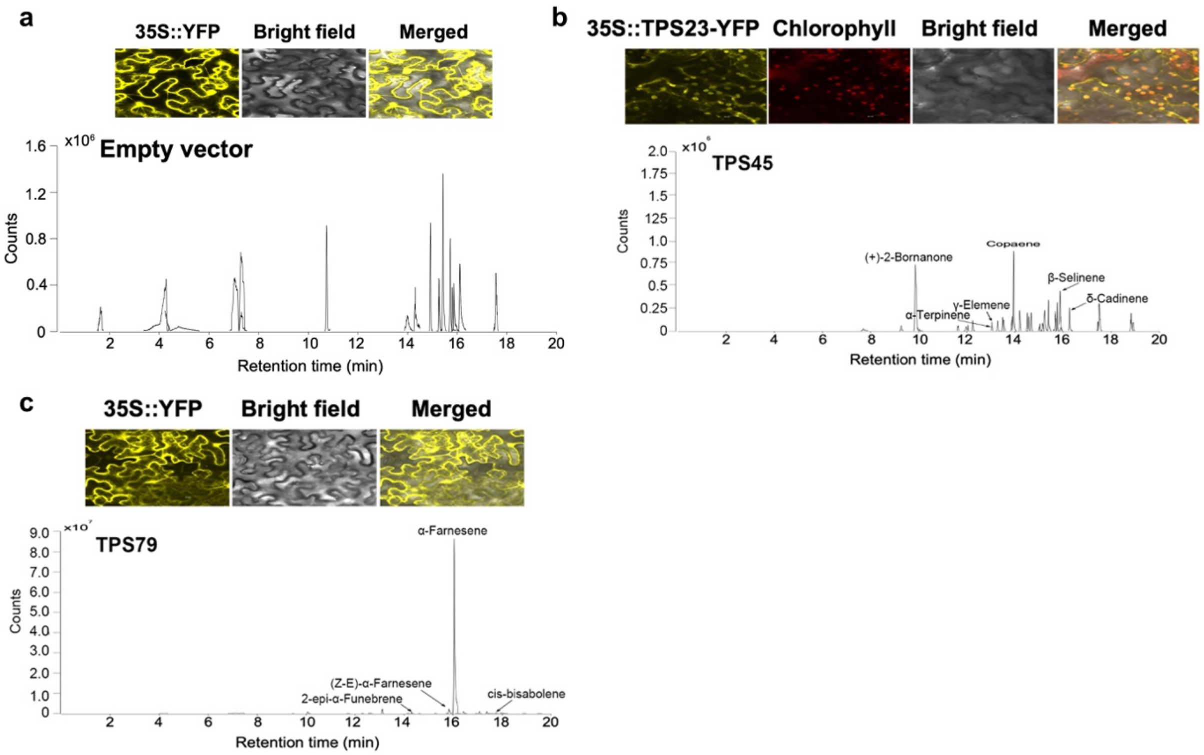

Fig. S24. Terpenoid biosynthesis gene characterization in *Cinnamomum camphora*. (a) Subcellular localization and transient function validation of *TPS-YFP* in tobacco. (b) Subcellular localization and transient function validation of *C. camphora TPS45* in tobacco. (c) Subcellular localization and transient function validation of *C. camphora TPS79* in tobacco. Each group of up panels are images for subcellular localization of TPS-YFP fusion proteins in tobacco mesophyll cells. Each bottom panel is the chromatogram of volatile compounds detected in tobacco leaves by GC-MS analysis.

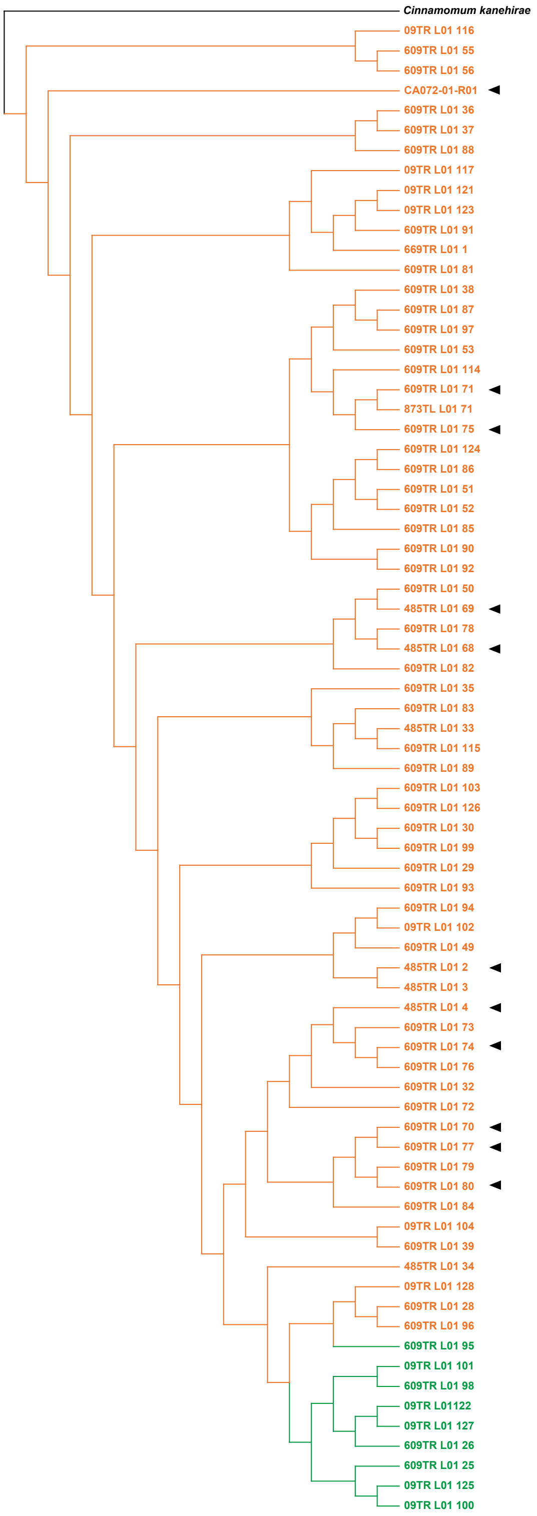

Fig. S25. The phylogenetic tree constructed by representative single nucleotide polymorphisms (SNPs) obtained from Chr 1 of 75 resequencing *Cinnamomum camphora* accessions using *C. kanehirae* as the outgroup. Branches and accession IDs in green represent samples in group I, and those in orange represent samples in group II in Fig. 4a and Fig. 4b. Individuals of *C. camphora* greater than 100 years old are marked by black triangles.

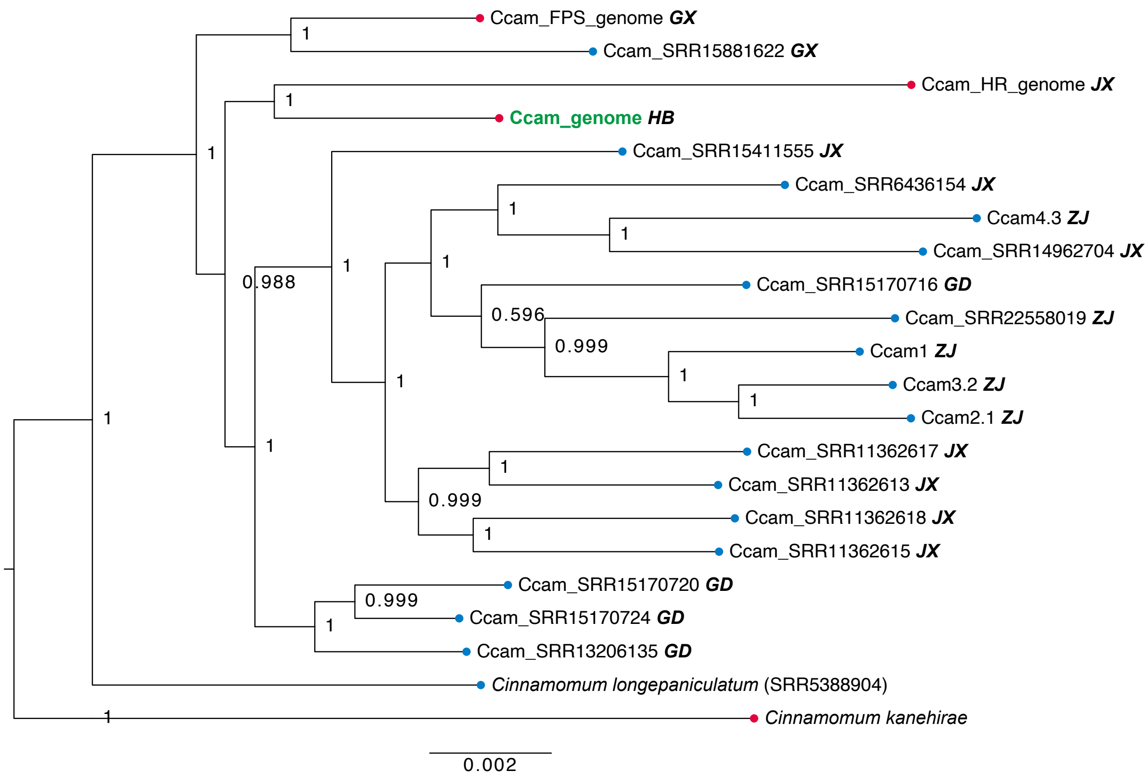

Fig. S26. The species tree based on amino acid sequences of identified single-copy orthogroups with a coalescent-based method from 22 samples with *Cinnamomum kanehirae* as the outgroup. Samples marked with red dots using genome files and ones with blue dots using *de novo* assembled transcriptome files. Bold capital letters indicate the sample collection site. GX represents Guangxi Province, JX represents Jiangxi Province, HB represents Hubei Province, ZJ represents Zhejiang Province, GD represents Guangdong Province.

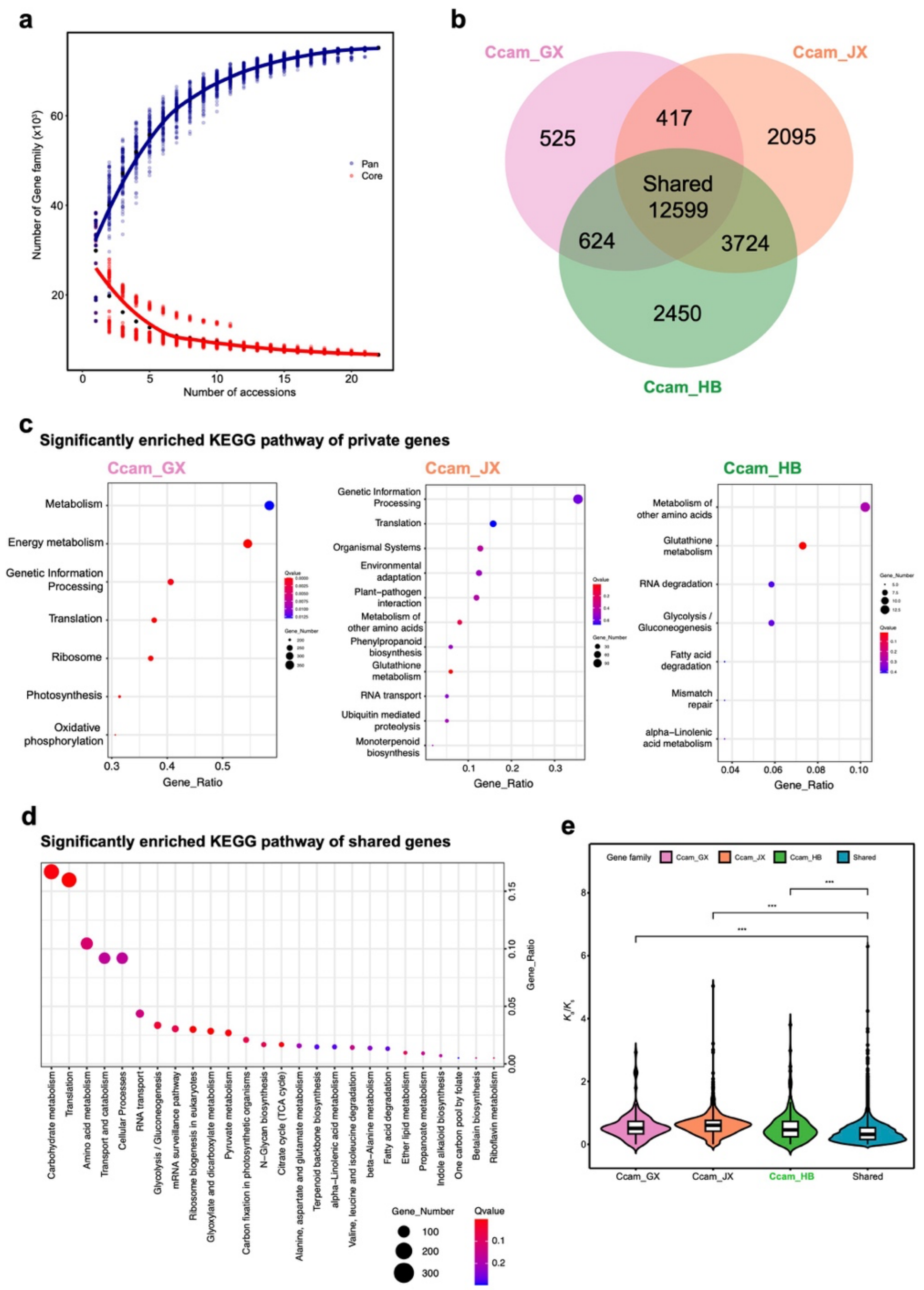

Fig. S27. Functional comparison analyses of *Cinnamomum camphora* genomes with high quality. (a) The number of pan-gene and core-gene families estimated based on pairwise gene family comparisons in 22 accessions (four genomes of *C. camphora,* one genome of *C. kanehirae*, one transcriptome of *C. longepaniculatum*, and 17 transcriptomes of *C. camphora*). Each black dot corresponds to a pan- or core-gene family size estimated by a particular combination. (b) A Venn analysis to obtain the private and shared genes of three *C. camphora* genomes. (c) KEGG enrichments of private genes in three *C. camphora* genomes. (d) KEGG enrichments of shared genes in three *C. camphora* genomes. (e) Comparisons of the K_a/K_s values of the private and shared genes in three *C. camphora* genomes.
